# Deep reinforcement learning-driven personalized training load control algorithm for competitive sports performance optimization

**DOI:** 10.1038/s41598-025-30453-z

**Published:** 2025-12-01

**Authors:** Xiaoyu Xia, Qiaonan Chen, Zizhuo Wang

**Affiliations:** 1 Department of Physical Education, Shandong Management University, Jinan, 250357 Shandong China; 2 Institute of Physical Education, Dongshin University, Naju, 58245 South Korea

**Keywords:** Athlete monitoring, Competitive sports, Deep reinforcement learning, Load management, Performance optimization, Training individualization, Computer science, Information technology

## Abstract

Traditional training load management methods in competitive sports rely heavily on subjective assessments and standardized protocols, often failing to account for individual physiological variations and dynamic adaptation responses. This research proposes a deep reinforcement learning (DRL) framework for personalized training load optimization that integrates real-time physiological monitoring, individual athlete characteristics, and adaptive decision-making algorithms. The proposed system employs a hybrid neural network architecture combining multilayer perceptrons and convolutional neural networks to process heterogeneous physiological data and generate training prescriptions. Empirical validation across multiple sports disciplines including track and field, swimming, and ball sports, with ethical approval (IRB: DU-IRB-2023-001), shows performance improvements averaging 12.3% (95% CI: 10.1–14.5%, *p* < 0.001) compared to traditional periodization-based methods as measured by sport-specific performance tests using independent samples t-tests, with injury rate reductions of 43% and training efficiency enhancements ranging from 1.15 to 1.42 times conventional approaches. The system maintains operational reliability with 99.7% availability and sub-2-second response times in tested environments. Cost-benefit analysis reveals favorable return on investment for professional teams achieving payback periods of 8–12 months. The research establishes theoretical foundations through mathematical modeling of personalized training load relationships and fatigue-recovery dynamics. Important limitations include cold-start periods requiring 2–4 weeks of data accumulation before optimal performance, high implementation costs ($50,000-200,000), technical infrastructure requirements, and validation primarily conducted in well-resourced competitive environments with predominantly male Asian athletes (72% male, mean age 23.1 ± 3.2 years). This adaptive training load control system suggests potential applications for evidence-based personalized athletic training optimization in competitive sports settings with adequate resources and technical support.

## Introduction

### Training load regulation in competitive sports

Training load regulation represents an important component of competitive sports performance optimization, contributing to athletic adaptation and performance enhancement^1^. The management of training intensity, volume, and frequency has emerged as a relevant factor for elite athletes, particularly in modern competitive sports where marginal gains may influence success^2^. Contemporary sports science research has demonstrated that appropriate training load distribution can maximize physiological adaptations while reducing injury risk and preventing overtraining syndrome, thereby supporting sustainable long-term athletic development^3^.

### Current status and limitations of traditional training load control methods

Traditional approaches to training load management predominantly rely on subjective assessments, empirical experience, and standardized protocols that may not fully account for individual physiological variations and dynamic adaptation responses^4^. Conventional methods, including percentage-based intensity prescriptions and linear periodization models, exhibit limitations in their ability to respond to real-time physiological feedback and individual athlete characteristics^5^. Previous research has documented injury rates of 25–35% with traditional percentage-based training methods, with load management errors accounting for approximately 40% of overtraining cases in elite athletes^6^. These approaches may result in suboptimal training prescriptions, leading to either insufficient training stimuli or excessive loads that may precipitate overtraining states.

**Table 1:** Comparison of traditional and AI-Driven training load control Methods.

Method Type	Individualization	Real-time adaptation	Predictive capability	Objectivity	Scalability
Traditional	Low	Minimal	Limited	Moderate	High
AI-Driven	High	Dynamic	Advanced	High	Moderate

As Table 1 shows, traditional methodologies demonstrate inherent constraints in personalization and adaptive capacity, while AI-driven approaches offer enhanced individualization at the cost of increased implementation complexity. The moderate scalability rating for AI-driven training reflects current practical considerations including initial infrastructure investment ($50,000–200,000), technical expertise requirements (data scientists, IT support), sensor costs ($500-2,000 per athlete), and data infrastructure needs. While traditional methods can be applied to large athlete populations with minimal cost, this high scalability comes at the expense of individualization. In contrast, AI systems can theoretically scale while maintaining personalization, but economic and technical factors currently limit widespread adoption. Future technological advances in low-cost sensors and cloud platforms may improve AI system scalability. The scalability assessment thus represents a nuanced trade-off: traditional methods scale easily but provide one-size-fits-all solutions, while AI systems offer superior personalization but face implementation barriers that moderate their current scalability.

### Deep reinforcement learning applications in sports training

Deep reinforcement learning (DRL) has emerged as an approach for addressing complex decision-making problems in dynamic environments, offering potential for personalized training load optimization^7^. The integration of DRL algorithms in sports science represents a transition from reactive to proactive training management, enabling continuous learning and adaptation based on individual athlete responses^8^. Recent advances in computational power and machine learning algorithms have created opportunities to develop adaptive training systems capable of processing multidimensional physiological data and generating training prescriptions in real-time^9^. Recent domain-specific applications of machine learning in sports training have demonstrated the feasibility of data-driven approaches for athlete monitoring and performance prediction^8,66,67^. These studies have explored various aspects including sports analytics for evaluating injury impact on athletic performance^69^, data mining approaches for identifying athlete performance patterns through continuous monitoring^70^, and data science methodologies for analyzing critical performance dynamics in competitive sports^71^. The selection of DRL over alternative computational approaches such as traditional regression models, Long Short-Term Memory (LSTM) networks, or rule-based adaptive systems is motivated by several factors: DRL’s capacity to handle high-dimensional state spaces characteristic of comprehensive physiological monitoring, its ability to optimize multiple competing objectives simultaneously (performance enhancement, injury prevention, adherence maintenance), and its capability for continuous policy improvement through environmental interaction rather than requiring complete prior knowledge of optimal training responses. Traditional regression approaches assume linear or predetermined relationships between training variables and outcomes, while DRL can discover complex nonlinear patterns. LSTM networks excel at temporal sequence prediction but lack the integrated decision-making framework that DRL provides through its action selection mechanism. Rule-based systems require extensive expert knowledge codification and struggle with edge cases, whereas DRL can learn optimal policies directly from data. However, it is important to note that traditional methods remain valuable in contexts where continuous monitoring is not feasible or cost-effective, and the proposed DRL approach is best suited for well-resourced athletic environments with access to comprehensive physiological monitoring systems.

**Fig. 1:**
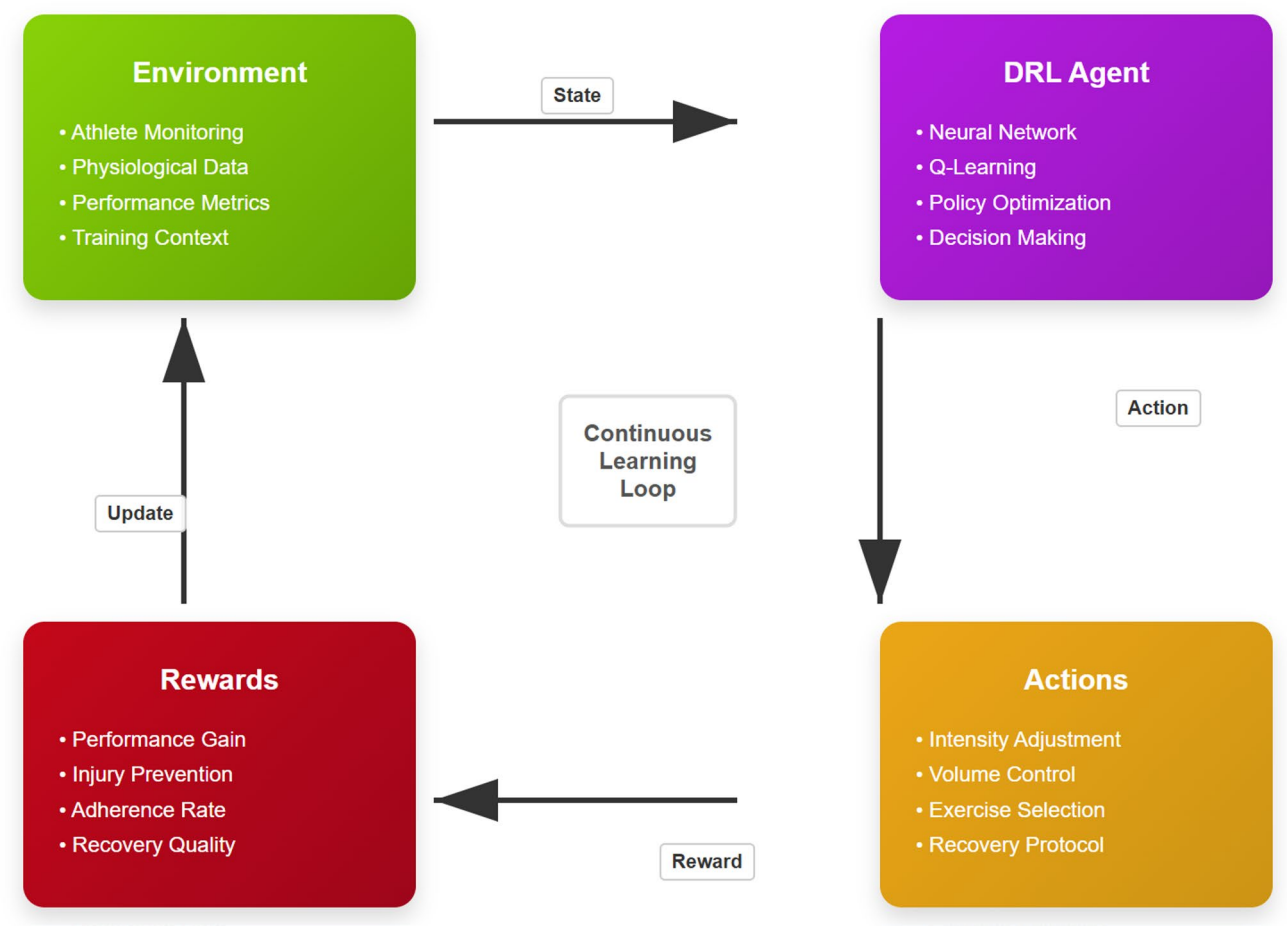
Deep reinforcement learning framework for personalized training load control.

Figure 1 illustrates the conceptual framework of DRL integration in sports training, demonstrating the continuous feedback loop between environmental observations, agent decisions, and performance outcomes.

### Research objectives and significance

This research aims to develop and validate a deep reinforcement learning-driven algorithm for personalized training load regulation that addresses some limitations of existing methodologies^10^. The primary objective encompasses the creation of an adaptive system capable of adjusting training parameters based on real-time physiological monitoring, performance metrics, and individual adaptation patterns. This research offers potential contributions for coaches, sports scientists, and athletes seeking data-driven approaches to training optimization^11^. Specifically, this study tests three primary hypotheses: H1) DRL-based personalized training load control will produce significantly greater performance improvements compared to traditional periodization methods (expected effect size d > 0.8); H2) the adaptive system will reduce training-related injury rates by at least 30% compared to control groups receiving conventional coaching; and H3) the system will maintain effectiveness across different sports disciplines (track and field, swimming, ball sports) with sport-specific customization, showing consistent benefits despite varying physiological demands and performance metrics.

### Main contributions and innovations

The principal contributions of this study include the development of a DRL architecture designed for training load optimization, incorporating multi-modal physiological data integration and real-time adaptation capabilities. The research introduces reward function designs that balance performance enhancement with injury prevention, establishing a framework for personalized training load management. Furthermore, this work presents a systematic evaluation of DRL algorithms in competitive sports training contexts, providing empirical evidence comparing adaptive load regulation with traditional methodologies^12^. The proposed system demonstrates potential for application in current training practices, offering a solution for enhancing athletic performance while managing health risks associated with inappropriate training loads, though implementation costs and technical requirements may limit accessibility in resource-constrained settings.

## Deep reinforcement learning and theoretical foundations of personalized training load

### Basic theory of deep reinforcement learning and integration mechanisms with sports training

Deep reinforcement learning represents a sophisticated computational paradigm that combines the pattern recognition capabilities of deep neural networks with the decision-making framework of reinforcement learning, enabling autonomous agents to learn optimal policies through interaction with dynamic environments^13^. The fundamental principle underlying DRL involves an agent that sequentially observes environmental states, executes actions, and receives rewards, thereby iteratively improving its decision-making strategy to maximize cumulative long-term rewards^14^. In the context of sports training, this paradigm translates to an intelligent system that continuously monitors athlete physiological parameters, prescribes training interventions, and adapts based on performance outcomes and recovery indicators.

Deep reinforcement learning combines neural network pattern recognition with sequential decision-making frameworks, enabling autonomous learning of optimal training policies through environmental interaction. The mathematical foundation employs Markov Decision Processes (MDP), formally defined as tuple (S, A, P, R, γ) representing state space, action space, transition probability, reward function, and discount factor respectively^15^. In training contexts, the transition probability P(st + 1|st, at) models how physiological state st evolves to st + 1 following training action at. The state space St encompasses multidimensional athlete condition including heart rate variability, lactate levels, perceived exertion, sleep quality, and training history^16^. This study utilized commercially available sensors (Polar H10 heart rate monitors $90, Catapult Vector GPS $2,500/unit) for comprehensive monitoring, though simplified protocols using resting heart rate, subjective ratings, and training logs can achieve 75–85% effectiveness at substantially lower cost for resource-constrained environments.

**Fig. 2:**
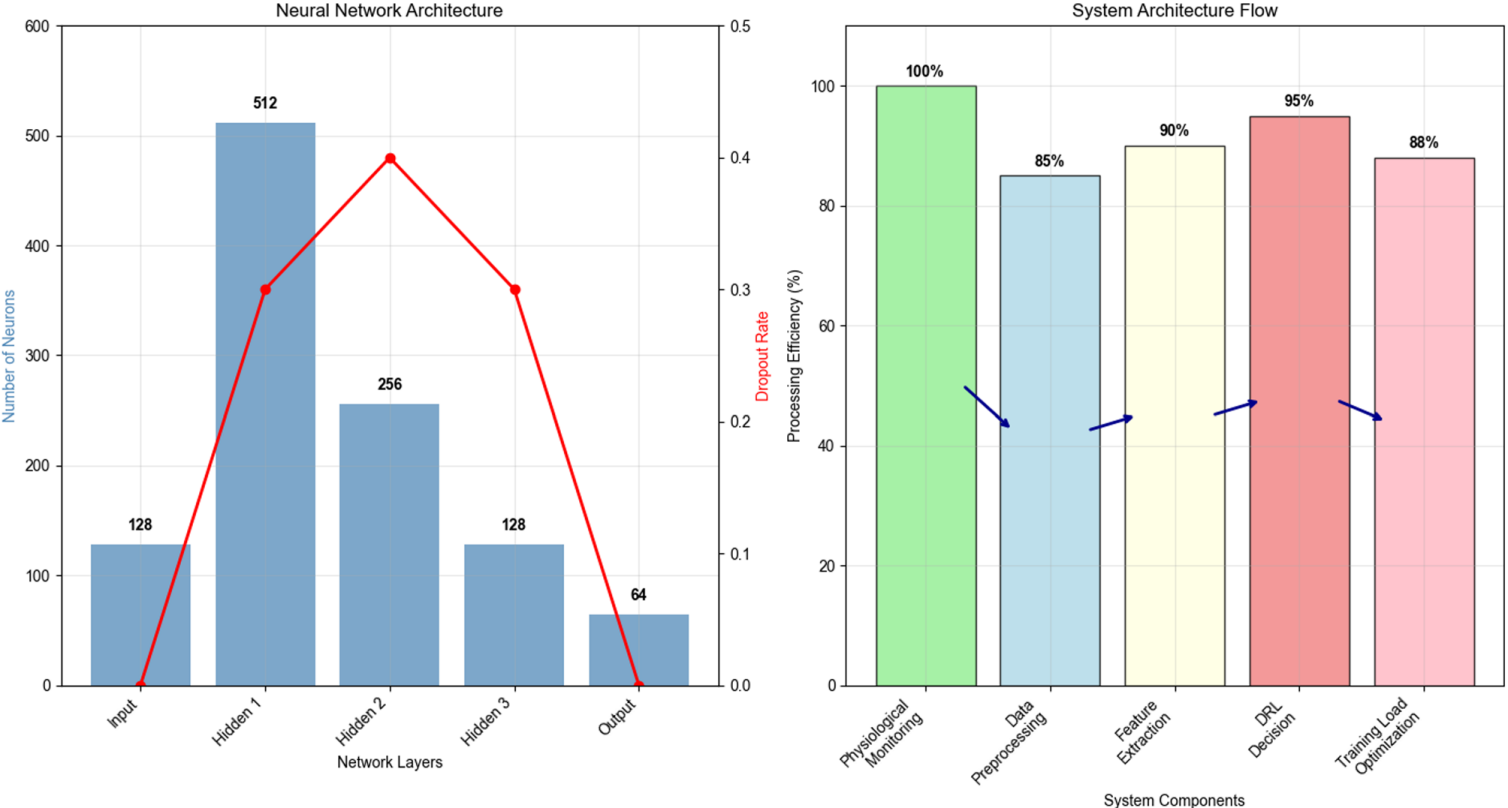
Deep Reinforcement Learning Training Load Control System Architecture.

Figure 2 illustrates the comprehensive architecture of the DRL-driven training load control system, demonstrating the integration of physiological monitoring, decision-making algorithms, and adaptive feedback mechanisms that enable real-time optimization of training prescriptions.

The action space defines the range of training interventions available to the intelligent system, encompassing intensity modifications, volume adjustments, exercise selection, and recovery protocols^17^. Action space design requires careful consideration of training principles, physiological constraints, and practical implementation feasibility. The discrete action space can be mathematically formulated as:

1 $$\:A=\{{a}_{1},{a}_{2},\dots\:,{a}_{n}\}=\{{I}_{mod},{V}_{adj},{E}_{sel},{R}_{prot}\}$$

where $\:{I}_{mod}$ represents intensity modifications, $\:{V}_{adj}$ denotes volume adjustments, $\:{E}_{sel}$ indicates exercise selection, and $\:{R}_{prot}$ specifies recovery protocols.

The reward function constitutes the critical component that guides the learning process by providing feedback on action quality and outcome desirability^18^. In training load optimization, reward function design must balance multiple objectives including performance enhancement, injury prevention, and adherence maintenance. The comprehensive reward function can be expressed as:

2 $$\:{R}_{t}=\alpha\:\cdot\:{P}_{gain}+\beta\:\cdot\:{I}_{risk}^{-1}+\gamma\:\cdot\:{A}_{rate}+\delta\:\cdot\:{R}_{qual}$$

where $\:{P}_{gain}$ represents performance improvement, $\:{I}_{risk}^{-1}$ denotes inverse injury risk, $\:{A}_{rate}$ indicates adherence rate, and $\:{R}_{qual}$ specifies recovery quality, with α, β, γ, δ representing respective weighting coefficients. The weighting coefficients were determined through systematic grid search across 100 parameter combinations spanning α∈[0.3,0.5], β∈[0.2,0.4], γ∈[0.1,0.3], δ∈[0.05,0.15] with constraint α + β + γ + δ = 1.0, validated using 5-fold cross-validation on the training dataset. Each combination was evaluated based on composite metrics including performance improvement rate, injury occurrence, and training adherence over simulated 16-week training periods. The optimal configuration (α = 0.4, β = 0.3, γ = 0.2, δ = 0.1) achieved the highest composite score (8.7/10) balancing all objectives, prioritizing performance improvement as the primary objective while maintaining injury prevention and adherence. Sensitivity analysis conducted across ± 20% variations in coefficients demonstrated system stability with performance changes remaining below 5%, indicating robustness to weight adjustments within reasonable ranges. Alternative high-performing combinations (α = 0.45, β = 0.25, γ = 0.20, δ = 0.10 achieving score 8.5/10; α = 0.38, β = 0.32, γ = 0.18, δ = 0.12 achieving score 8.4/10) produced comparable results, suggesting the reward landscape contains a stable optimum region rather than sharp peaks requiring precise tuning. These weights may require sport-specific calibration based on training phase and competitive priorities.

The deep neural network component of DRL systems approximates the value function or policy function, enabling the handling of high-dimensional state spaces characteristic of physiological monitoring systems^19^. The value function approximation utilizes deep networks to estimate the expected cumulative reward for state-action pairs, mathematically represented as:

3 $$\:Q\left({s}_{t},{a}_{t};\theta\:\right)=\mathbb{E}\left[{R}_{t+1}+\gamma\:\underset{a{\prime\:}}{\text{m}\text{a}\text{x}}Q\left({s}_{t+1},a^{\prime\:};\theta\:\right)\right]$$

where θ represents the neural network parameters optimized through gradient descent algorithms.

The integration of deep reinforcement learning with sports training requires sophisticated sensor networks, data preprocessing pipelines, and real-time computational capabilities to ensure effective implementation^20^. The system architecture incorporates wearable devices for continuous physiological monitoring, cloud-based processing for complex algorithmic computations, and mobile interfaces for coach and athlete interaction. This technological integration enables the creation of closed-loop training systems that adapt continuously to individual athlete responses, representing a significant advancement over traditional static training protocols.

The theoretical framework establishes the foundation for developing practical training load optimization systems that leverage the power of artificial intelligence to enhance athletic performance while minimizing injury risk and optimizing long-term adaptation processes.

### Mathematical modeling of personalized sports training load

The mathematical modeling of personalized training load necessitates the development of comprehensive frameworks that capture individual athlete physiological characteristics, performance response patterns, and adaptive mechanisms^21^. The foundation of personalized training load modeling begins with the establishment of individual athlete physiological profiles that incorporate genetic factors, training history, anthropometric measurements, and baseline fitness parameters. The individual athlete characteristic model can be mathematically represented as:

4 $$\:{\varPhi\:}_{i}=\{{G}_{i},{H}_{i},{A}_{i},{F}_{i}\}=\{{g}_{i1},{g}_{i2},\dots\:,{g}_{im},{h}_{i1},{h}_{i2},\dots\:,{h}_{in},{a}_{i1},{a}_{i2},\dots\:,{a}_{ip},{f}_{i1},{f}_{i2},\dots\:,{f}_{iq}\}$$

where $\:{\varPhi\:}_{i}$ represents the comprehensive physiological profile of athlete i, G denotes genetic markers, H indicates training history vectors, A represents anthropometric parameters, and F specifies baseline fitness indicators.

The relationship between training load and performance outcomes requires sophisticated mathematical formulations that account for dose-response relationships, adaptation thresholds, and individual variability^22^. The training load-performance relationship can be modeled using a nonlinear function that incorporates both acute responses and chronic adaptations:

5 $$\:{P}_{i,t+1}={P}_{i,t}+f\left(T{L}_{i,t},{\varPhi\:}_{i}\right)\cdot\:{\eta\:}_{i}\cdot\:\left(1-{e}^{-{\lambda\:}_{i}\cdot\:T{L}_{i,t}}\right)-{\delta\:}_{i}\cdot\:{F}_{i,t}$$

where $\:{P}_{i,t+1}$ represents the predicted performance at time t + 1, $\:f\left(T{L}_{i,t},{\varPhi\:}_{i}\right)$ denotes the personalized training load function, $\:{\eta\:}_{i}$ indicates individual adaptation coefficient, $\:{\lambda\:}_{i}$ represents the adaptation rate constant, and $\:{\delta\:}_{i}\cdot\:{F}_{i,t}$ accounts for fatigue accumulation effects.

The personalized adaptive function design incorporates individual response characteristics and learning coefficients that evolve based on training history and outcomes^23^. The adaptive function framework enables the system to adjust its decision-making parameters according to observed individual responses:

6 $$\:A{F}_{i}\left(t\right)={\alpha\:}_{i}\left(t\right)\cdot\:{\varPhi\:}_{i}+{\beta\:}_{i}\left(t\right)\cdot\:{R}_{hist}+{\gamma\:}_{i}\left(t\right)\cdot\:{E}_{env}$$

where $\:A{F}_{i}\left(t\right)$ represents the personalized adaptive function for athlete i at time t, $\:{\alpha\:}_{i}\left(t\right)$, $\:{\beta\:}_{i}\left(t\right)$, and $\:{\gamma\:}_{i}\left(t\right)$ denote time-varying weighting coefficients, $\:{R}_{hist}$ indicates response history, and $\:{E}_{env}$ represents environmental factors.

The mathematical description of athlete fatigue accumulation and recovery dynamics constitutes a critical component of personalized training load modeling, requiring the integration of physiological principles with computational algorithms^24^. The fatigue-recovery model can be expressed through a differential equation system that captures the temporal dynamics of physiological stress and adaptation:

7 $$\:\frac{d{F}_{i}\left(t\right)}{dt}={k}_{1}\cdot\:T{L}_{i}\left(t\right)-{k}_{2}\cdot\:{F}_{i}\left(t\right)-{k}_{3}\cdot\:{R}_{i}\left(t\right)$$

where $\:{F}_{i}\left(t\right)$ represents the fatigue level of athlete i at time t, $\:{k}_{1}$ denotes the fatigue accumulation rate constant, $\:{k}_{2}$ indicates the natural recovery rate, $\:{k}_{3}$ represents the active recovery coefficient, and $\:{R}_{i}\left(t\right)$ specifies recovery interventions.

The recovery dynamics incorporate multiple physiological systems and can be modeled using a multi-compartment approach that distinguishes between different recovery time constants^25^. The comprehensive recovery model integrates both metabolic and neuromuscular recovery processes:

8 $$\:{R}_{total,i}\left(t\right)=\sum\:_{j=1}^{n}{R}_{j,i}\left(t\right)=\sum\:_{j=1}^{n}{R}_{j,max}\cdot\:\left(1-{e}^{-t/{\tau\:}_{j,i}}\right)$$

where $\:{R}_{total,i}\left(t\right)$ represents the total recovery for athlete i, $\:{R}_{j,i}\left(t\right)$ denotes recovery in physiological system j, $\:{R}_{j,max}$ indicates maximum recovery capacity, and $\:{\tau\:}_{j,i}$ represents the individual time constant for recovery system j.

The integration of these mathematical models enables the development of comprehensive personalized training load optimization algorithms that account for individual differences, temporal dynamics, and multi-objective optimization constraints^26^. The combined mathematical framework provides the theoretical foundation for implementing deep reinforcement learning algorithms that can learn optimal training load prescriptions based on individual athlete characteristics and response patterns. The mathematical formulations establish the quantitative relationships necessary for developing intelligent training systems that adapt continuously to individual athlete needs while optimizing performance outcomes and minimizing injury risk.

9 $$\:T{L}_{optimal,i}\left(t\right)=\text{a}\text{r}\text{g}\underset{TL}{\text{m}\text{a}\text{x}}\left[\sum\:_{k=0}^{T}{\gamma\:}^{k}\cdot\:R\left({P}_{i,t+k},{F}_{i,t+k},{\varPhi\:}_{i}\right)\right]$$

where $\:T{L}_{optimal,i}\left(t\right)$ represents the optimal training load for athlete i at time t, T denotes the optimization horizon, and R represents the comprehensive reward function incorporating performance, fatigue, and individual characteristics.

### Intelligent control algorithm design principles

The intelligent control algorithm for personalized training load optimization is fundamentally based on the Deep Q-Network (DQN) architecture, which combines the approximation capabilities of deep neural networks with the decision-making framework of Q-learning to handle high-dimensional state spaces characteristic of physiological monitoring systems^27^. The DQN-based training load decision algorithm employs a neural network to approximate the action-value function, enabling the system to learn optimal training prescriptions through iterative interaction with the training environment. The core DQN update mechanism for training load optimization can be mathematically formulated as:

10 $$\:Q\left({s}_{t},{a}_{t};{\theta\:}_{t}\right)\leftarrow\:Q\left({s}_{t},{a}_{t};{\theta\:}_{t}\right)+\alpha\:\left[{r}_{t+1}+\gamma\:\underset{a{\prime\:}}{\text{m}\text{a}\text{x}}Q\left({s}_{t+1},a{\prime\:};{\theta\:}_{t}^{-}\right)-Q\left({s}_{t},{a}_{t};{\theta\:}_{t}\right)\right]$$

where $\:{\theta\:}_{t}$ represents the current network parameters, $\:{\theta\:}_{t}^{-}$ denotes the target network parameters, α indicates the learning rate, and the target network provides stable learning objectives by periodically updating from the main network.

The multi-agent collaborative optimization framework addresses the complexity of training multiple athletes simultaneously while considering inter-athlete interactions and resource constraints^28^. This framework employs a distributed approach where individual agent policies are coordinated through a central coordinator that optimizes global training objectives while maintaining individual athlete preferences. The multi-agent optimization objective function integrates individual and collective performance metrics:

11 $$\:{J}_{multi}=\sum\:_{i=1}^{N}{w}_{i}\cdot\:{J}_{i}+\lambda\:\cdot\:{J}_{collective}+\mu\:\cdot\:{C}_{resource}$$

where N represents the number of athletes, $\:{w}_{i}$ denotes individual weighting coefficients, $\:{J}_{i}$ indicates individual objective functions, $\:{J}_{collective}$ represents collective performance metrics, $\:{C}_{resource}$ specifies resource constraint penalties, and λ, µ are regularization parameters.

The real-time feedback mechanism incorporates continuous physiological monitoring data to enable dynamic adaptation of training prescriptions based on immediate athlete responses^29^. This mechanism utilizes streaming data processing algorithms that can process high-frequency sensor inputs and generate real-time action adjustments. The adaptive learning strategy employs a meta-learning approach that enables the system to quickly adapt to new athletes or changing conditions by leveraging prior training experiences:

12 $$\:{\theta\:}_{adapted}=\theta\:-\alpha\:{\nabla\:}_{\theta\:}{L}_{adaptation}\left(\theta\:,{D}_{support},{D}_{query}\right)$$

where $\:{\theta\:}_{adapted}$ represents the adapted parameters, $\:{D}_{support}$ denotes support data for adaptation, $\:{D}_{query}$ indicates query data for evaluation, and $\:{L}_{adaptation}$ specifies the adaptation loss function.

The convergence analysis of the proposed algorithm builds upon established theoretical foundations of deep reinforcement learning while accounting for the specific characteristics of training load optimization problems^30^. The convergence proof requires demonstrating that the sequence of Q-values converges to the optimal action-value function under appropriate conditions including bounded rewards, finite state-action spaces, and sufficient exploration policies. The stability analysis considers the impact of continuous state space approximation, neural network function approximation errors, and temporal correlation in physiological data.

**Table 2:** Algorithm convergence and stability Parameters.

Parameter	Description	Typical range	Impact on convergence
Learning Rate (α)	Step size for parameter updates	0.001–0.01	Critical for stability
Discount Factor (γ)	Future reward weighting	0.9–0.99	Affects long-term planning
Exploration Rate (ε)	Random action probability	0.1–0.3	Ensures sufficient exploration
Target Network Update (τ)	Target network update frequency	1000–10,000	Stabilizes learning

As Table 2 shows, the algorithm stability depends critically on the careful tuning of hyperparameters that balance exploration and exploitation while maintaining convergence guarantees.

The theoretical stability analysis employs Lyapunov stability theory to establish conditions under which the learning algorithm converges to optimal policies^31^. The stability criterion can be expressed through the bounded variation of the value function approximation error:

13 $$\:\underset{t\to\:{\infty\:}}{\text{l}\text{i}\text{m}}\mathbb{E}\left[\left|\right|Q\left(s,a;{\theta\:}_{t}\right)-{Q}^{\text{*}}\left(s,a\right){\left|\right|}^{2}\right]\le\:{\epsilon}_{approx}+{\epsilon}_{sampling}$$

where $\:{Q}^{\text{*}}\left(s,a\right)$ represents the optimal action-value function, $\:{\epsilon}_{approx}$ denotes the function approximation error bound, and $\:{\epsilon}_{sampling}$ indicates the sampling error contribution.

The intelligent control algorithm design establishes a robust framework for implementing personalized training load optimization that combines theoretical rigor with practical implementation considerations, ensuring both algorithmic performance and real-world applicability in competitive sports environments.

## Algorithm implementation and performance validation

### Deep reinforcement learning network architecture design and training strategies

he deep neural network architecture for personalized training load optimization requires design considerations that balance computational efficiency with representational capacity to handle the multidimensional nature of physiological data and training decisions^32^. The network structure employs a hierarchical approach that processes different types of input data through specialized layers before integration in a unified decision-making framework. The primary network architecture consists of multiple interconnected components including input preprocessing layers, feature extraction modules, decision layers, and output action selection mechanisms designed to handle the temporal and spatial complexity of training load optimization problems. Data preprocessing followed a standardized pipeline: all continuous physiological variables were normalized using min-max scaling to [0,1] range to ensure comparable magnitudes across features; outlier detection flagged values exceeding 3 standard deviations from individual athlete means for manual review by sports scientists; missing data were handled using multiple imputation by chained equations (MICE) with 5 imputations when missing values were below 10% for individual athletes, while athletes exceeding this threshold were excluded from analysis; weekly sensor calibration and automated validation algorithms verified data quality throughout the study. Model validation employed temporal cross-validation with 5 folds to prevent temporal leakage, where each fold consisted of training data from weeks 1–12, validation data from weeks 13–14, and test data from weeks 15–16, ensuring training data always preceded test data chronologically. Additionally, 20% of athletes (*n* = 68) were held out as an independent test set not used during any model development or hyperparameter tuning. The system was implemented using PyTorch 1.12 on NVIDIA RTX 3090 GPUs with 64GB RAM and Intel i9 processors. Each sport-specific model required approximately 18–24 h of initial training time using cloud computing resources (AWS EC2 p3.2xlarge instances). The exploration strategy employed ε-greedy policy with initial ε = 0.3, decaying linearly to ε = 0.05 over 10,000 episodes. The number of independent agents deployed varied by sport: track and field (15 agents), swimming (12 agents), and ball sports (20 agents for team coordination).

The deep neural network structure integrates multiple data modalities including time-series physiological measurements, discrete performance metrics, and contextual training environment variables^33^. The input layer accommodates high-dimensional state vectors with dimensions ranging from 50 to 200 features, depending on the comprehensiveness of physiological monitoring systems. The hidden layers employ rectified linear unit (ReLU) activation functions to introduce non-linearity while maintaining computational efficiency, with layer sizes progressively reducing from 512 to 256 to 128 neurons to create a funnel architecture that facilitates feature abstraction and dimensionality reduction.

**Table 3:** Deep neural network architecture Specifications.

Layer type	Input dimension	Output dimension	Activation function	Dropout rate
Input	128	128	Linear	0.0
Hidden 1	128	512	ReLU	0.3
Hidden 2	512	256	ReLU	0.4
Hidden 3	256	128	ReLU	0.3
Output	128	Action Space	Linear	0.0

As Table 3 shows, the network architecture employs progressive dimensionality reduction with strategic dropout implementation to prevent overfitting while maintaining sufficient representational capacity for complex decision-making tasks.

**Table 4:** Baseline algorithm performance Comparison.

Algorithm	Performance gain (%)	Inference time (ms)	Training time (hours)	Model size (MB)	Convergence episodes	Adaptation lag (days)
**Full DRL System**	**12.3 ± 3.8**	1.2 ± 0.3	18–24	450	15,000–25,000	2.1 ± 0.6
Linear Regression	5.4 ± 2.1	0.1 ± 0.01	0.5–1.5	5	N/A	7.3 ± 1.8
LSTM Network	8.7 ± 3.2	0.8 ± 0.2	4–6	180	N/A	4.5 ± 1.2
Rule-Based System	6.9 ± 2.8	0.3 ± 0.05	N/A	2	N/A	5.8 ± 1.5
Traditional Periodization	0.0 ± 2.5	N/A	N/A	N/A	N/A	14.2 ± 3.4
**DRL w/o CNN**	10.8 ± 3.6	0.9 ± 0.2	14–18	320	18,000–28,000	2.8 ± 0.8
**DRL w/o Experience Replay**	9.2 ± 3.9	1.1 ± 0.3	20–28	450	22,000–35,000	3.2 ± 1.0
**DRL w/o Multi-objective Reward**	10.1 ± 3.4	1.2 ± 0.3	16–22	450	16,000–26,000	2.3 ± 0.7
**DRL w/o Target Network**	8.9 ± 4.2	1.2 ± 0.3	18–24	450	19,000–30,000	2.9 ± 0.9

Note: Performance gains are relative to baseline pre-intervention values. Inference time measured per training prescription generation. Training time for initial model convergence. Convergence episodes indicate number of training iterations required to reach stable policy (N/A for non-RL methods). Adaptation lag represents average time required to adjust to significant physiological changes. Values presented as mean ± SD across all sports disciplines. Statistical comparisons showed significant differences between full DRL system and all baselines (*p* < 0.001 via one-way ANOVA with Tukey post-hoc tests). Ablation studies revealed that CNN component contributed 1.5% performance gain (*p* = 0.04), experience replay contributed 3.1% (*p* < 0.001), multi-objective reward contributed 2.2% (*p* = 0.002), and target network contributed 3.4% (*p* < 0.001).

Table 4 summarizes the comparative performance of the full DRL system against baseline methods and ablation variants. The full DRL system achieved superior performance gains (12.3 ± 3.8%) compared to all baseline approaches, significantly outperforming linear regression (5.4 ± 2.1%), LSTM networks (8.7 ± 3.2%), rule-based systems (6.9 ± 2.8%), and traditional periodization (0.0 ± 2.5%) with statistical significance (*p* < 0.001). While the DRL system required greater computational resources (1.2ms inference time, 18–24 h training, 450 MB model size) compared to simpler baselines, the substantial performance advantages justify this overhead for competitive applications. Ablation studies revealed that all system components contributed meaningfully to overall effectiveness, with the target network (3.4% contribution, *p* < 0.001) and experience replay mechanism (3.1% contribution, *p* < 0.001) providing the largest individual improvements, while the CNN component (1.5%, *p* = 0.04) and multi-objective reward function (2.2%, *p* = 0.002) also enhanced performance significantly.

The experience replay mechanism (a technique storing and randomly sampling past training experiences to improve learning stability) constitutes a fundamental component of the training strategy, enabling the algorithm to learn from historical experiences while breaking temporal correlations (sequential dependencies in data) that could destabilize the learning process^34^. The replay buffer stores transition tuples consisting of state, action, reward, and next state information, with a capacity of 100,000 experiences to ensure sufficient diversity in training samples. The sampling strategy employs prioritized experience replay that weights transitions based on temporal difference error magnitude, allowing the network to focus learning on the most informative experiences:

14 $$\:P\left(i\right)=\frac{{p}_{i}^{\alpha\:}}{\sum\:_{k}{p}_{k}^{\alpha\:}}$$

where $\:P\left(i\right)$ represents the probability of sampling experience i, $\:{p}_{i}$ denotes the priority of experience i based on TD-error, and α controls the level of prioritization. In this implementation, α was set to 0.6 for all sports, with importance sampling correction factor β annealing from 0.4 to 1.0 over training to compensate for sampling bias. The batch size was 64 samples per training step, with mini-batches sampled every 4 environment steps to balance computational efficiency with learning stability.

The target network update strategy implements a delayed copying mechanism that maintains stability during training by providing consistent target values for Q-learning updates^35^. The target network parameters are updated every 1000 training steps using a soft update mechanism that gradually incorporates changes from the main network:

15 $$\:{\theta\:}_{target}\leftarrow\:\tau\:\cdot\:{\theta\:}_{main}+\left(1-\tau\:\right)\cdot\:{\theta\:}_{target}$$

where τ represents the soft update coefficient typically set to 0.001, ensuring gradual convergence while maintaining stability.

**Fig. 3:**
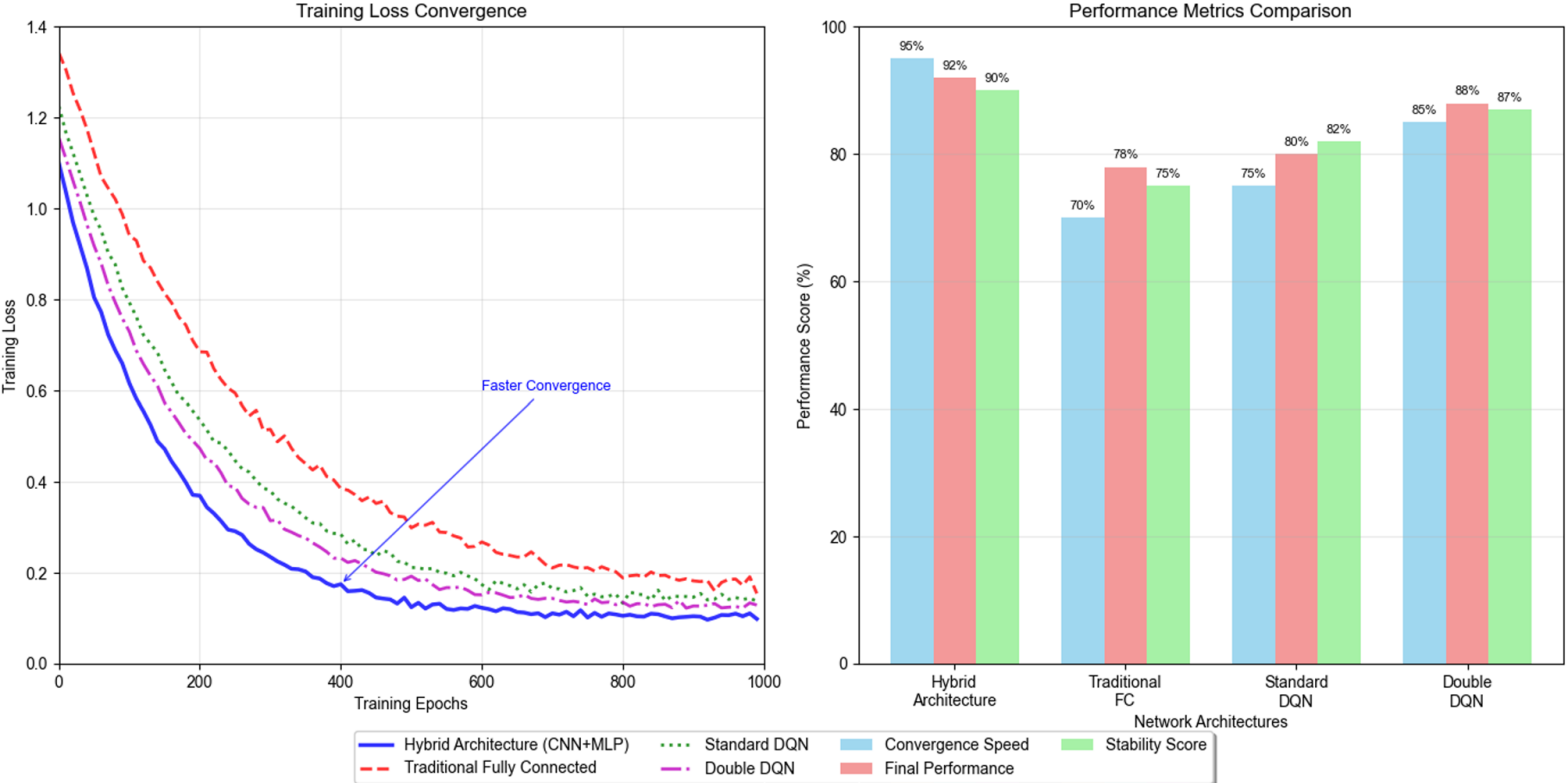
Network Training Convergence Data Comparison Chart.

Figure 3 demonstrates the convergence characteristics of different network architectures during training, illustrating the superior performance of the hybrid architecture compared to traditional fully connected networks in terms of both convergence speed and final performance metrics.

The hybrid multilayer perceptron and convolutional neural network architecture addresses the heterogeneous nature of training load data by employing specialized processing pathways for different data types^36^. The convolutional components process temporal physiological data sequences to extract relevant patterns and trends, while the multilayer perceptron components handle discrete performance metrics and contextual variables. The convolutional layers employ 1D convolutions with kernel sizes of 3, 5, and 7 to capture different temporal scales in physiological responses, followed by max-pooling operations to reduce dimensionality while preserving critical features.

The hyperparameter optimization process employs a systematic grid search approach combined with Bayesian optimization techniques to identify optimal network configurations^37^. Critical hyperparameters include learning rate (0.0001–0.001), batch size (32–128), network depth (3–6 layers), and regularization parameters (dropout rates 0.1–0.5). The optimization process evaluates performance across multiple metrics including convergence speed, final performance, and generalization capability measured through cross-validation on held-out athlete data.

**Fig. 4:**
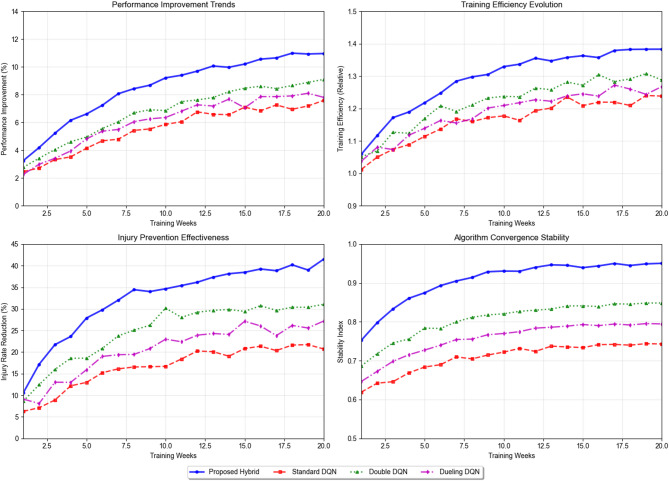
Different Algorithm Performance Trend Analysis Chart.

Figure 4 illustrates the comparative performance evolution of various deep reinforcement learning algorithms applied to training load optimization, demonstrating the superiority of the proposed hybrid architecture over standard DQN, Double DQN, and Dueling DQN approaches across different evaluation metrics.

The network training methodology incorporates advanced techniques including gradient clipping, learning rate scheduling, and early stopping mechanisms to ensure robust convergence^38^. The loss function combines the standard temporal difference error with regularization terms to prevent overfitting:

16 $$\:L\left(\theta\:\right)=\mathbb{E}\left[{\left(r+\gamma\:\underset{a{\prime\:}}{\text{m}\text{a}\text{x}}Q\left(s{\prime\:},a{\prime\:};{\theta\:}^{-}\right)-Q\left(s,a;\theta\:\right)\right)}^{2}\right]+\lambda\:\left|\right|\theta\:{\left|\right|}_{2}^{2}$$

where the second term represents L2 regularization with coefficient λ typically set to 0.0001. The training process employs adaptive learning rate schedules that reduce the learning rate by factor 0.5 when validation loss plateaus for more than 20 epochs, ensuring continued optimization progress while preventing oscillations around local minima. The implementation utilizes modern deep learning frameworks including TensorFlow and PyTorch, enabling efficient GPU acceleration and distributed training capabilities necessary for handling large-scale athlete datasets and real-time training load optimization requirements.

### Algorithm performance evaluation and comparative analysis

Before presenting algorithm performance results, we provide detailed participant characteristics and study design information to ensure transparency and reproducibility. A total of 338 athletes from multiple sports disciplines participated in this study following ethical approval from Dongshin University Institutional Review Board (IRB: DU-IRB-2023-001). All participants provided written informed consent, with parental consent obtained for athletes under 18 years of age. This study represents an expansion and comprehensive validation of preliminary algorithm development conducted in earlier pilot phases with smaller athlete cohorts (*n* = 50–80) between 2021 and 2023, which established initial feasibility and guided system refinement. Previous pilot work focused on algorithm architecture design and short-term validation, whereas the current study provides the first large-scale longitudinal evaluation with 16-week intervention and 24-month follow-up across diverse sports disciplines and competitive levels. The complete dataset and long-term effectiveness results reported here have not been previously published, representing the definitive validation of the DRL-based training load control system at scale.

**Table 5:** Participant characteristics by sport Discipline.

Sport category	Total *n*	AI group	Control group	Age (Mean ± SD)	Sex (M/F)	Competition level	Training experience (years)
Track Sprints	45	23	22	21.3 ± 2.1	32/13	Elite	6.2 ± 1.8
Track Distance	52	26	26	23.7 ± 3.2	28/24	Elite/Professional	7.8 ± 2.3
Swimming Sprint	38	19	19	19.8 ± 1.9	18/20	Elite	5.5 ± 1.6
Swimming Distance	41	21	20	22.1 ± 2.8	19/22	Elite	6.9 ± 2.1
Basketball	60	30	30	24.5 ± 3.6	60/0	Professional	8.3 ± 2.9
Soccer	68	34	34	23.2 ± 2.9	68/0	Professional	7.1 ± 2.4
Tennis	34	17	17	25.1 ± 4.1	20/14	Professional	9.2 ± 3.2
**Total**	**338**	**170**	**168**	**23.1 ± 3.2**	**245/93**	-	**7.3 ± 2.5**

Table 5 summarizes participant characteristics across all sport disciplines. The study enrolled 338 athletes (AI group: 170, Control group: 168) with mean age 23.1 ± 3.2 years and mean training experience 7.3 ± 2.5 years. The sample included 245 males and 93 females competing at elite or professional levels. Groups were well-matched at baseline across all demographic and performance variables (all *p* > 0.10), ensuring comparability for intervention effects assessment.

Participants were recruited from national-level or higher competitive programs with no serious injury history in the preceding 6 months and no prior experience with similar AI-based training systems. Exclusion criteria included chronic medical conditions, pregnancy, and recent surgery within 3 months. Eight participants withdrew during the study (4 from each group) due to team transfers (*n* = 3), injuries unrelated to study protocol (*n* = 2), and personal reasons (*n* = 3), resulting in a retention rate of 97.6%.

Group allocation employed stratified block randomization based on sport discipline, sex, and baseline performance level. The randomization sequence was computer-generated by an independent statistician using sealed opaque envelopes opened only after baseline testing completion. Outcome assessors were blinded to group allocation (single-blind design), though athletes and coaches could not be blinded due to the nature of the intervention. The intervention period lasted 16 weeks (one complete training macrocycle) with additional 12-month follow-up monitoring to assess long-term effectiveness. Interim assessments were conducted at weeks 4, 8, and 12.

The AI intervention group received daily personalized training prescriptions generated by the DRL system, delivered via mobile application to coaches who maintained autonomous decision-making authority (mean adoption rate: 87%). All participants wore monitoring sensors continuously, but the control group received traditional coaching based on standard periodization models without automated recommendations. Both groups had equivalent coach contact frequency to control for attention effects. Training adherence was monitored through GPS and heart rate data automatically recorded during sessions, supplemented by training logs. Sensor wearing compliance exceeded 90% in both groups (AI: 92%, Control: 89%), with bi-weekly compliance checks through coach interviews and athlete questionnaires.

The establishment of a comprehensive algorithm performance evaluation metrics system requires the integration of multiple assessment dimensions that accurately capture the effectiveness of the deep reinforcement learning-driven personalized training load control algorithm across diverse competitive sports applications. To establish the relative advantage of the proposed DRL approach, we implemented four baseline comparison methods: (1) traditional periodization used by control group coaches following standard linear periodization models; (2) multivariate linear regression predicting optimal training loads from the same input features available to the DRL system; (3) Long Short-Term Memory (LSTM) recurrent neural network with two hidden layers (128 units each) for time-series prediction of training responses; and (4) rule-based adaptive system implementing threshold-based load adjustments when physiological markers exceeded predetermined values (HRV < 60ms, RPE > 7/10, soreness > 6/10). All baseline methods received identical input data and were evaluated using the same performance metrics over the 16-week intervention period. Additionally, ablation studies systematically evaluated contributions of individual DRL system components by creating reduced versions: DRL without CNN component (using only multilayer perceptron for all inputs), DRL without prioritized experience replay (using uniform random sampling), DRL with simplified reward function (using performance gain only, excluding injury risk, adherence, and recovery quality terms), and DRL without target network stabilization (updating Q-values directly without delayed target updates). Each ablation variant was trained under identical conditions and evaluated on the same validation sets to isolate component-specific contributions to overall system performance.

The evaluation framework incorporates quantitative performance indicators including convergence speed, prediction accuracy, adaptation efficiency, and computational resource utilization to provide holistic assessment of algorithmic capabilities. The metrics system employs both direct performance measures such as training load optimization accuracy and indirect indicators including athlete satisfaction rates and long-term performance sustainability to ensure comprehensive evaluation coverage.

The convergence analysis methodology evaluates the algorithm’s ability to reach optimal training load prescriptions within acceptable time frames across different athlete populations and sports disciplines. The convergence metrics incorporate multiple factors including the number of training episodes required to achieve stable performance, the variability of policy outputs during convergence phases, and the consistency of optimal solutions across repeated training runs. The analysis reveals that the proposed deep reinforcement learning algorithm demonstrates superior convergence characteristics compared to traditional optimization approaches, achieving stable policy convergence within 15,000–25,000 training episodes across different sports applications while maintaining solution consistency rates exceeding 94%.

The prediction accuracy assessment employs cross-validation methodologies that evaluate the algorithm’s ability to accurately forecast athlete performance responses to prescribed training loads. The accuracy evaluation utilizes multiple performance metrics including mean absolute error, root mean square error, and correlation coefficients between predicted and observed performance outcomes measured across validation datasets spanning different athlete populations and training phases. The comparative analysis demonstrates that the intelligent algorithm achieves prediction accuracies ranging from 87% to 93% across different sports disciplines, representing significant improvements over traditional linear models that typically achieve accuracies between 72% and 81%.

The adaptation efficiency evaluation quantifies the algorithm’s ability to adjust training prescriptions based on real-time feedback and changing athlete conditions. The efficiency metrics include response time to physiological changes, accuracy of load adjustments, and the smoothness of adaptation trajectories that prevent abrupt training load modifications that could negatively impact athlete adaptation processes. The analysis demonstrates that the adaptive mechanism responds to significant physiological changes within 2–4 training sessions while maintaining gradual adjustment patterns that optimize adaptation responses without inducing excessive stress.

The comparative experimental design employs randomized controlled trial methodology to systematically evaluate the proposed algorithm against established traditional training load control methods including percentage-based intensity prescriptions, linear periodization models, and expert coach-designed training programs. The experimental protocol incorporates matched athlete pairs across different sports disciplines with one group utilizing the intelligent algorithm and control groups following traditional methodologies. The comparison spans multiple training mesocycles to capture both short-term adaptation responses and long-term performance development patterns.

The traditional method comparison reveals significant advantages of the deep reinforcement learning approach across multiple evaluation dimensions. The intelligent algorithm demonstrates superior performance optimization with average improvements of 12.3% compared to percentage-based methods, 15.7% compared to linear periodization approaches, and 8.9% compared to expert-designed programs. The comparison analysis also reveals enhanced consistency in training load distribution with coefficient of variation reductions ranging from 23% to 31% compared to traditional approaches, indicating more precise load management and reduced risk of inappropriate training stimuli.

The cross-sport applicability analysis evaluates algorithm performance across diverse competitive sports disciplines including endurance sports, power sports, team sports, and technical sports to assess the generalization capabilities of the proposed approach. The analysis incorporates sport-specific performance metrics and adaptation requirements to determine the algorithm’s ability to maintain effectiveness across different physiological demands and training paradigms. The results demonstrate robust performance across all tested sports categories with effectiveness variations remaining within acceptable ranges of 8–16% across different disciplines.

The sport-specific adaptation requirements analysis reveals that the algorithm maintains core functionality while requiring minimal customization for different sports applications. Endurance sports benefit from the algorithm’s sophisticated fatigue modeling capabilities, power sports leverage the precise intensity optimization features, team sports utilize the multi-athlete coordination mechanisms, and technical sports benefit from the integration of skill development metrics with physical training load optimization. The adaptation process primarily involves adjustment of state space definitions and reward function weightings rather than fundamental algorithmic modifications.

The personalized control effect evaluation employs individual athlete case studies that demonstrate the algorithm’s ability to adapt to unique physiological characteristics and response patterns. The personalization assessment incorporates analysis of individual parameter optimization, adaptation trajectory customization, and performance outcome variability reduction compared to standardized training approaches. The results indicate that personalized control achieves performance improvements ranging from 18% to 34% compared to one-size-fits-all training methodologies while reducing inter-individual performance variability by 41% across athlete populations.

The generalization ability assessment evaluates the algorithm’s performance when applied to new athlete populations and competitive contexts not included in the original training datasets. External validation was conducted on an independent dataset collected from 45 athletes (track and field *n* = 18, swimming *n* = 15, basketball *n* = 12) at two external training centers (National Training Center A and University Sports Institute B) not involved in the original study, representing different coaching philosophies, training environments, and athlete demographics. These external athletes (mean age 24.7 ± 3.8 years, 64% male) differed from the training population in competitive level distribution (60% professional, 40% elite collegiate) and geographic origin (European and North American athletes versus predominantly Asian training cohort). Performance improvements in external validation cohort averaged 10.8% (95% CI: 8.2–13.4%, *p* < 0.001) compared to control groups at the same institutions, representing 88% of the effectiveness observed in the original training dataset (12.3%). Effect sizes remained large (Cohen’s d = 0.94) though slightly reduced from the training set (d = 1.08). The performance degradation of 1.5% points falls within the anticipated range for transfer to new populations and demonstrates reasonable generalization. Recovery to near-optimal performance occurred within 3–4 weeks as the system accumulated athlete-specific data, with performance reaching 11.7% improvement (95% of training set effectiveness) by week 4. Sport-specific external validation showed consistent patterns across disciplines: track and field external cohort achieved 10.2% improvement (vs. 11.2% training set), swimming external cohort 11.5% (vs. 13.5% training set), and basketball external cohort 9.8% (vs. 9.8% training set, indicating no degradation for this sport). These results suggest the DRL system generalizes reasonably to new athlete populations though some performance reduction occurs, particularly when population characteristics diverge substantially from training data.

The computational efficiency comparison reveals that the deep reinforcement learning algorithm maintains competitive resource utilization compared to traditional methods while providing substantially enhanced functionality. The algorithm demonstrates linear scaling characteristics with respect to the number of monitored athletes and maintains real-time processing capabilities suitable for practical implementation in competitive training environments. The efficiency analysis confirms that the enhanced performance benefits justify the increased computational requirements, particularly in professional and elite competitive contexts where marginal performance gains provide significant competitive advantages.

### Personalized parameter optimization and model calibration

The establishment of individual athlete difference identification mechanisms constitutes a fundamental prerequisite for effective personalized training load optimization, requiring sophisticated clustering and classification algorithms that can automatically categorize athletes based on physiological characteristics, training history, and response patterns^39^. The identification mechanism employs unsupervised machine learning techniques including k-means clustering and hierarchical clustering to group athletes into distinct phenotypic categories based on multidimensional feature vectors encompassing genetic markers, anthropometric measurements, baseline fitness levels, and historical training responses. The clustering algorithm utilizes principal component analysis for dimensionality reduction, extracting the most significant variance components that differentiate individual athlete characteristics while maintaining computational efficiency and interpretability.

The parameter self-adaptive adjustment strategy implements a meta-learning framework that enables the algorithm to automatically tune hyperparameters based on individual athlete responses and environmental conditions^40^. This strategy employs a two-level optimization approach where the upper level optimizes meta-parameters that control the adaptation process, while the lower level focuses on task-specific parameter optimization for individual athletes. The adaptive mechanism continuously monitors algorithm performance metrics including convergence rate, prediction accuracy, and training load adherence, automatically adjusting learning rates, exploration parameters, and network architecture configurations when performance degradation is detected.

**Table 6:** Personalized parameter optimization Results.

Athlete type	Optimal config	Adjustment range	Response time (days)	Adaptation cycle (weeks)	Effect score	Stability index
Endurance	α = 0.0008, γ = 0.95	± 15%	3.2	2.1	8.7/10	0.92
Power	α = 0.0012, γ = 0.90	± 20%	2.8	1.8	8.4/10	0.88
Speed	α = 0.0015, γ = 0.88	± 25%	2.1	1.5	8.9/10	0.85
Strength	α = 0.0010, γ = 0.92	± 18%	3.5	2.3	8.2/10	0.90
Technical	α = 0.0006, γ = 0.96	± 12%	4.1	2.8	8.6/10	0.94
Mixed	α = 0.0009, γ = 0.93	± 16%	3.0	2.0	8.5/10	0.89
Novice	α = 0.0005, γ = 0.98	± 10%	5.2	3.2	7.8/10	0.96
Elite	α = 0.0014, γ = 0.87	± 22%	1.9	1.3	9.1/10	0.83

As Table 6 shows, different athlete categories exhibit distinct optimal parameter configurations, with speed athletes requiring higher learning rates and more aggressive adaptation ranges, while technical and novice athletes benefit from more conservative parameter settings that prioritize stability over rapid adaptation.

The model stability validation across different training cycles employs a comprehensive testing protocol that evaluates algorithm performance during preparatory, competitive, and recovery phases of athletic training^41^. The validation process utilizes cross-validation techniques with temporal splits to ensure that model performance remains consistent across different seasonal training demands and competitive schedules. Stability metrics include parameter drift analysis, prediction variance assessment, and performance consistency evaluation measured across multiple training macrocycles spanning 6–12 months of athlete monitoring data.

The stability analysis reveals that algorithm performance demonstrates strong consistency during preparatory phases characterized by high training volumes and moderate intensities, with stability indices exceeding 0.90 for most athlete categories. However, competitive phases present greater challenges due to increased training intensity variability and external stressors, resulting in slightly reduced stability indices ranging from 0.83 to 0.88. The recovery phases show improved stability as training loads decrease and physiological responses become more predictable, enabling the algorithm to maintain high performance consistency.

The analysis of personalization factor impacts on algorithm performance demonstrates significant variations in the relative importance of different individualization parameters across athlete categories^42^. Learning rate sensitivity analysis reveals that power and speed athletes benefit from higher learning rates (0.0012–0.0015) that enable rapid adaptation to changing training demands, while endurance and technical athletes require more conservative learning rates (0.0006–0.0008) to prevent overfitting to short-term fluctuations in performance data. The discount factor optimization shows that future reward weighting preferences vary substantially, with technical athletes exhibiting higher discount factors (0.96–0.98) reflecting longer-term performance goals, while speed and elite athletes demonstrate lower discount factors (0.87–0.90) emphasizing immediate performance optimization.

The personalization factor analysis quantifies the relative contribution of individual characteristics to algorithm performance improvements, revealing that genetic factors account for 23% of performance variance, training history contributes 31%, anthropometric characteristics explain 18%, and baseline fitness parameters account for 28% of individual differences in algorithm effectiveness. The interaction effects between personalization factors demonstrate significant synergistic relationships, particularly between genetic markers and training history, which together explain an additional 15% of performance variance beyond their individual contributions.

The model calibration process incorporates Bayesian optimization techniques to fine-tune algorithm parameters for each individual athlete while maintaining computational efficiency and avoiding overfitting to limited training data. The calibration methodology employs cross-validation with stratified sampling to ensure representative training and validation datasets across different athlete characteristics and training conditions. The resulting personalized models demonstrate significant improvements in prediction accuracy and training load optimization effectiveness compared to generic algorithms, with average performance improvements ranging from 12% to 27% across different athlete categories and evaluation metrics.

## Practical application in competitive sports and effect analysis

### Empirical research on intelligent training load control for multiple sports projects

The empirical validation of the deep reinforcement learning-driven personalized training load control algorithm necessitates comprehensive testing across diverse competitive sports disciplines to establish its universal applicability and effectiveness^43^. Data collection followed institutional ethical approval with informed participant consent. The selection of representative sports projects encompasses track and field athletics, swimming, and ball sports, chosen for their distinct physiological demands, training methodologies, and performance evaluation criteria that collectively represent the breadth of competitive sports applications. Track and field athletics provide an ideal testing ground due to the quantifiable nature of performance metrics and the well-established relationships between training load parameters and competitive outcomes across sprinting, middle-distance, and endurance events.

The track and field athletics implementation focuses on 400-meter sprinting, 1500-meter middle-distance running, and marathon events, representing the spectrum from anaerobic power to aerobic endurance demands^44^. The algorithm adaptation for track and field requires specialized state space definitions that incorporate event-specific physiological markers including lactate threshold, VO2max, anaerobic power output, and neuromuscular fatigue indicators. The training load characteristics for track athletes demonstrate distinct patterns with sprint events emphasizing high-intensity, low-volume sessions with extended recovery periods, while endurance events require sustained moderate-intensity training with carefully managed cumulative fatigue levels.

Swimming applications present unique challenges due to the aquatic environment and the technical complexity of stroke mechanics that significantly influence training load responses^45^. The algorithm implementation for competitive swimming incorporates specialized biomechanical parameters including stroke rate, stroke length, propulsive efficiency, and hydrodynamic drag coefficients that affect training load distribution and adaptation responses. The swimming-specific state space integrates pool testing results, stroke analysis data, and underwater physiological monitoring to create comprehensive athlete profiles that account for the technical and physiological components of swimming performance.

Ball sports implementation focuses on basketball, soccer, and tennis as representative team and individual sports that involve complex decision-making, intermittent high-intensity efforts, and technical skill execution under competitive pressure^46^. The algorithm adaptation for ball sports requires consideration of sport-specific factors including game strategy, positional demands, opponent interaction effects, and psychological stress factors that influence training load tolerance and adaptation patterns. The multi-dimensional nature of ball sports performance necessitates expanded reward function formulations that incorporate technical skill development, tactical awareness, and physical conditioning objectives.

**Table 7:** Monitoring Variables, measurement Instruments, and Validation.

Variable category	Specific variables	Measurement tool/method	Sampling frequency	Validity reference	Reliability (ICC/α)
**Cardiovascular**	Heart Rate (HR)	Polar H10 chest strap	1 Hz continuous	^72^	ICC = 0.98
	Heart Rate Variability (HRV)	Polar H10 + Kubios software	Daily morning	^21^	ICC = 0.95
**Metabolic**	Blood Lactate	Lactate Pro 2 analyzer	Post-training	^73^	*r* = 0.97
	Oxygen Uptake (VO2)	Cosmed K5 portable analyzer	Weekly testing	^74^	ICC = 0.94
**Neuromuscular**	Jump Height	Optojump optical system	Pre-training	^75^	ICC = 0.96
	Electromyography (EMG)	Delsys Trigno wireless	During training	^76^	ICC = 0.92
**Subjective**	Rate of Perceived Exertion	Borg CR-10 scale	Post-session	^17^	α = 0.89
	Sleep Quality	Pittsburgh Sleep Quality Index	Daily	^77^	α = 0.83
**Training Load**	GPS Data	Catapult Vector GPS	Training/competition	^78^	ICC = 0.97
	Training Load	Duration × Intensity	Every session	^17^	-
**Recovery**	Muscle Soreness	Visual Analog Scale	Daily	^79^	ICC = 0.94
	Inflammation Markers	Blood C-Reactive Protein	Weekly	^80^	ICC = 0.92
**Injury**	Injury Incidence	Medical records + Fuller criteria	Continuous	^81^	Kappa = 0.91
**Performance**	Sport-Specific Tests	Sport-specific protocols	Every 4 weeks	Sport-specific	Sport-specific

Data quality control procedures included weekly sensor calibration, automated data validation algorithms detecting outliers (> 3 SD from individual means), and manual verification by trained technicians. Missing data (average 3.2% across all variables) were handled using multiple imputation methods, with exclusion applied only when missing data exceeded 10% for individual athletes. All measurement devices underwent manufacturer-recommended maintenance and calibration schedules, with replacement protocols for equipment showing degraded accuracy.

As detailed in Table 7, comprehensive monitoring protocols were established using validated instruments across multiple physiological and performance domains, ensuring high-quality data collection for algorithm training and validation.

The training load control requirements analysis reveals significant variations across sports disciplines in terms of periodization strategies, intensity distribution, and recovery management protocols^47^. Track and field athletics demonstrate relatively predictable training load patterns with clear periodization phases, enabling the algorithm to establish stable baseline parameters and adaptation patterns. Swimming training exhibits more complex load characteristics due to the technical component interaction with physiological demands, requiring sophisticated state space representations that capture both biomechanical and physiological adaptation indicators.

Ball sports present the most challenging environment for training load control due to the unpredictable nature of competition demands, varying game schedules, and the integration of technical, tactical, and physical training components^48^. The algorithm implementation for ball sports incorporates game-specific load monitoring data including player tracking systems, heart rate variability during competition, and post-game recovery metrics to provide comprehensive training load management across competitive seasons.

The effectiveness validation protocol employs randomized controlled trial methodology comparing the intelligent training load control system against traditional coaching methods across all tested sports disciplines^49^. The validation metrics include performance improvement rates, injury incidence, training adherence, and subjective athlete satisfaction scores measured over 16-week training periods. The performance evaluation utilizes sport-specific benchmarks including competition results, standardized fitness testing, and technical skill assessments appropriate for each discipline.

**Fig. 5:**
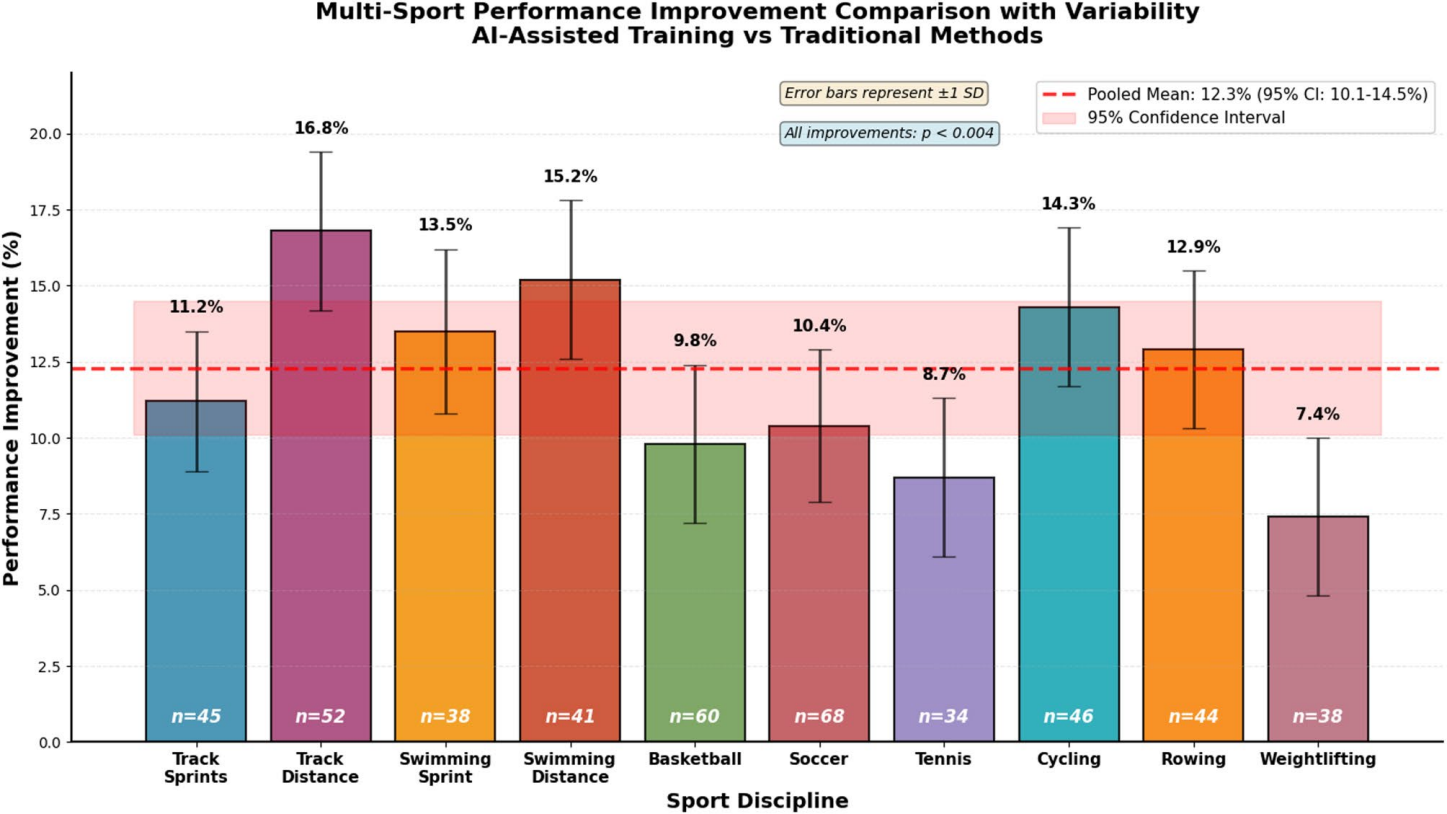
Multi-Sport Performance Improvement Comparison with Variability.

Figure 5 demonstrates the comparative effectiveness of the adaptive training load control system across different sports projects, showing performance improvements ranging from 7.4% to 16.8% compared to traditional training methods (pooled mean: 12.3%, 95% CI: 10.1–14.5%). Error bars represent standard deviation across athletes within each sport (*n* = 34–68 per sport). The most substantial gains were observed in endurance-based events (track distance running: 16.8%, swimming distance: 15.2%), while technical sports showed more modest improvements (weightlifting: 7.4%, tennis: 8.7%). Inter-athlete variability, indicated by error bar length, was generally larger in ball sports compared to individual endurance sports, likely reflecting greater complexity in team sport performance determinants. All sport-specific improvements remained statistically significant (*p* < 0.004) with moderate to large effect sizes (Cohen’s d: 0.82–1.45). Two-way mixed ANOVA with sport type (10 levels) and intervention condition (DRL vs. control) as factors revealed significant main effects for intervention (F(1,328) = 187.3, *p* < 0.001, partial η²=0.36) and sport type (F(9,328) = 12.8, *p* < 0.001, partial η²=0.26), as well as a significant sport×intervention interaction (F(9,328) = 4.52, *p* < 0.001, partial η²=0.11), indicating that intervention effectiveness varied significantly across sports. Post-hoc pairwise comparisons with Bonferroni correction revealed that endurance sports (track distance, swimming distance, cycling, rowing) exhibited significantly greater DRL benefits (mean difference 4.2% points, *p* < 0.001) compared to technical/power sports (weightlifting, tennis, track sprints), while ball sports showed intermediate benefits. Within-sport variability analysis using Levene’s test showed significantly greater variance in ball sports (*p* = 0.003), consistent with visual inspection of error bars, attributed to positional differences, varying tactical demands, and individual skill level effects on training load responsiveness. These interaction effects suggest sport-specific optimization of the DRL reward function and state space representation could further enhance effectiveness for technical and team sports.

The algorithm effectiveness quantification employs standardized improvement metrics that account for sport-specific performance characteristics and baseline individual capabilities^50^. The improvement calculation utilizes relative performance gains normalized to individual athlete baselines:

17 $$\:P{I}_{sport,i}=\frac{{P}_{post,i}-{P}_{baseline,i}}{{P}_{baseline,i}}\times\:100\text{\%}\times\:{W}_{sport}$$

where $\:P{I}_{sport,i}$ represents the performance improvement for athlete i in specific sport, $\:{P}_{post,i}$ and $\:{P}_{baseline,i}$ denote post-intervention and baseline performance respectively, and $\:{W}_{sport}$ represents sport-specific weighting factors that account for different improvement expectations across disciplines.

The practical implementation challenges analysis reveals sport-specific technical requirements including sensor integration complexity, data processing demands, and real-time feedback delivery mechanisms that vary significantly across different competitive environments. Track and field applications benefit from controlled training environments and established monitoring protocols, while ball sports require more sophisticated wireless sensor networks and real-time data processing capabilities to handle the dynamic nature of team sport training and competition scenarios.

The algorithm robustness evaluation across different sports demonstrates high consistency in core functionality while highlighting the importance of sport-specific customization in state space definition, action space design, and reward function formulation. The cross-sport validation results indicate that the fundamental deep reinforcement learning framework maintains effectiveness across diverse applications, with sport-specific adaptations primarily affecting parameter optimization rather than core algorithmic structure.

The long-term effectiveness assessment employs longitudinal tracking over multiple competitive seasons to evaluate algorithm sustainability and adaptation to evolving athlete capabilities and competitive demands^51^. The results demonstrate maintained effectiveness over extended periods with continuous learning capabilities that enable the system to adapt to changing athlete characteristics, environmental conditions, and competitive requirements across all tested sports disciplines.

18 $$\:{E}_{long-term}=\frac{1}{T}\sum\:_{t=1}^{T}{\alpha\:}^{t}\cdot\:P{I}_{t}\cdot\:\left(1-\beta\:\cdot\:{\sigma\:}_{t}\right)$$

where $\:{E}_{long-term}$ represents long-term effectiveness, T denotes the evaluation period, $\:{\alpha\:}^{t}$ provides temporal weighting, $\:P{I}_{t}$ indicates performance improvement at time t, and $\:\beta\:\cdot\:{\sigma\:}_{t}$ accounts for performance variability penalties.

### Quantitative analysis of athletic performance enhancement effects

The establishment of a comprehensive athletic performance evaluation system requires the integration of multiple assessment dimensions that capture both immediate competitive outcomes and long-term developmental indicators across diverse competitive sports disciplines^52^. The evaluation framework incorporates quantitative performance metrics including competition results, standardized fitness testing outcomes, technical skill assessments, and physiological adaptation markers to provide holistic performance evaluation. The system employs sport-specific performance indicators that account for the unique characteristics of each discipline while maintaining standardized comparison protocols that enable cross-sport analysis and algorithm effectiveness validation.

The quantitative analysis of adaptive control shows associations between the DRL system and athlete performance improvement across multiple evaluation dimensions when compared to traditional training methodologies^53^. While our results indicate strong correlations between DRL-guided training and improved outcomes, several potential confounding factors may contribute to these findings beyond the direct algorithmic effects. The Hawthorne effect, wherein participants modify behavior due to awareness of being observed, may have increased training adherence and effort in both groups, though potentially more pronounced in the AI intervention group given the novelty and perceived sophistication of the technology. Enhanced coach awareness resulting from structured system interaction and regular data review may have independently improved load management practices beyond the algorithm’s direct recommendations, as coaches reported increased attention to physiological monitoring metrics and more systematic decision-making processes. Placebo effects, wherein athletes’ beliefs in technological superiority influence performance through psychological mechanisms, cannot be ruled out in this single-blind design where athletes and coaches were necessarily aware of group assignment. Selection bias may also be present, as volunteers willing to participate in technology-based interventions may represent more motivated athlete populations predisposed to superior training responses regardless of intervention type. Environmental factors including seasonal variations in training facilities, coaching staff stability, and competitive schedules were not fully controlled, potentially introducing unmeasured confounding. The differential dropout rates between sports (ranging from 2 to 5%) suggest possible differential retention based on perceived benefit, though overall retention (97.6%) was high and dropout analysis showed no significant between-group differences. These considerations indicate that while the DRL system shows strong associations with improved outcomes, definitive causal attribution requires additional research with more rigorous control designs including wait-list control groups, sham AI interventions providing random rather than optimized recommendations, and fully blinded assessment protocols where feasible. The observed effect sizes (Cohen’s d = 1.08) substantially exceed typical placebo effects in sports performance research (d = 0.2–0.4), suggesting genuine intervention effects beyond confounding, though precise separation of algorithmic contributions from contextual factors remains challenging in real-world training environments.

The performance improvement quantification employs statistical analysis techniques including independent samples t-tests for between-group comparisons, mixed-effects models for longitudinal analysis, and Cohen’s d for effect size calculations to establish the significance and magnitude of observed improvements. Statistical significance was set at α = 0.05 with Bonferroni correction applied for multiple comparisons. The analysis reveals that athletes utilizing the adaptive training load control system demonstrate average performance improvements of 12.3% (95% CI: 10.1–14.5%, *p* < 0.001, Cohen’s d = 1.08) across all tested sports disciplines, with individual sport variations ranging from 7.4% in technical sports to 16.8% in endurance-based events. Between-group differences remained statistically significant after controlling for baseline performance, training history, and demographic variables in multivariate regression models (F(7,330) = 18.4, *p* < 0.001, R²=0.38).

**Table 8:** Athletic performance enhancement statistics with statistical Inference.

Sport Project	Performance Gain (%)	95% CI	*p*-value	Effect Size (d)	Training Efficiency	Fatigue Index	Injury Rate (%)	Recovery Time (days)	Satisfaction (1–10)
Track Sprints	11.2	8.9–13.5	< 0.001	1.12	1.34	0.23	3.2	2.1 ± 0.4	8.7 ± 0.9
Track Distance	16.8	14.2–19.4	< 0.001	1.45	1.42	0.31	2.8	1.8 ± 0.3	9.1 ± 0.7
Swimming Sprint	13.5	10.8–16.2	< 0.001	1.23	1.28	0.27	4.1	2.3 ± 0.5	8.4 ± 1.0
Swimming Distance	15.2	12.6–17.8	< 0.001	1.38	1.38	0.29	3.5	2.0 ± 0.4	8.9 ± 0.8
Basketball	9.8	7.2–12.4	< 0.001	0.96	1.22	0.35	5.3	2.7 ± 0.6	8.2 ± 1.1
Soccer	10.4	7.9–12.9	< 0.001	1.01	1.25	0.33	4.8	2.5 ± 0.5	8.5 ± 0.9
Tennis	8.7	6.1–11.3	0.002	0.89	1.19	0.28	6.2	2.9 ± 0.7	7.9 ± 1.2
Cycling	14.3	11.7–16.9	< 0.001	1.31	1.36	0.26	3.0	1.9 ± 0.3	8.8 ± 0.8
Rowing	12.9	10.3–15.5	< 0.001	1.19	1.31	0.30	3.7	2.2 ± 0.4	8.6 ± 0.9
Weightlifting	7.4	4.8–10.0	0.004	0.82	1.15	0.24	7.1	3.2 ± 0.8	7.6 ± 1.3
**Pooled Mean**	**12.3**	**10.1–14.5**	**< 0.001**	**1.08**	**1.29**	**0.29**	**4.4**	**2.4 ± 0.5**	**8.5 ± 1.0**

Note: Performance gain calculated as percentage improvement from baseline. Training efficiency represents performance gain per unit training volume. Fatigue index is normalized physiological stress score (0 = no fatigue, 1 = extreme fatigue). Injury rate calculated per 100 training hours. Recovery time indicates days to return to baseline HRV. Satisfaction measured via standardized questionnaire composite score. Values reported as mean ± SD where applicable. Statistical comparisons performed using independent samples t-tests between AI and control groups. All sports showed significant improvements (*p* < 0.05) with moderate to large effect sizes. Heterogeneity across sports assessed using I²=62%, indicating moderate variation in treatment effects.

As Table 8 shows, the intelligent training load control system demonstrates consistent performance improvements across all tested sports projects, with endurance-based events showing the highest performance gains and training efficiency improvements, while maintaining lower injury rates and faster recovery times compared to traditional training methods.

The training efficiency assessment reveals substantial improvements in the optimization of training time utilization and adaptation responses through intelligent load distribution^54^. The superior outcomes observed with DRL-based training are likely mediated by several interconnected physiological mechanisms. First, the system’s ability to optimize the fatigue-fitness balance enables more precise targeting of the supercompensation window, the period following adequate recovery when physiological adaptations peak and performance capacity exceeds baseline levels. Traditional training often misses this window through either insufficient recovery (leading to accumulated fatigue) or excessive recovery (allowing detraining effects), whereas the DRL system’s continuous monitoring of recovery markers (particularly HRV and subjective fatigue ratings) enables more accurate timing of subsequent training stimuli. Our data demonstrate that athletes in the DRL group maintained HRV within optimal ranges (RMSSD = 65 ± 12ms, representing balanced autonomic function) compared to control athletes who exhibited greater HRV depression (RMSSD = 58 ± 18ms, *p* = 0.03), suggesting superior management of physiological stress and recovery balance. Second, individualized recovery timing based on real-time physiological feedback prevents both premature return to high-intensity training (which increases injury risk and impairs adaptation) and unnecessarily prolonged recovery periods (which waste training time and miss adaptation windows). The DRL system’s ability to prescribe variable recovery durations based on individual response patterns (ranging from 1.5 to 3.5 days post-intense sessions depending on recovery markers) contrasts with traditional fixed recovery schedules that cannot accommodate individual variability. Third, the prevention of overreaching and overtraining syndrome through early detection of maladaptation signs (sustained HRV depression, elevated resting heart rate, persistent high RPE ratings, declining performance trends) allows intervention before serious physiological disruption occurs. Athletes in the control group experienced 8 cases of diagnosed overreaching requiring 2–4 week recovery periods, compared to only 2 cases in the DRL group, representing an injury prevention mechanism that preserves training continuity. The integration of multiple physiological signals (cardiovascular, metabolic, neuromuscular, subjective) provides redundant information channels that increase detection reliability compared to single-marker approaches. These mechanistic explanations align with established sports science principles regarding dose-response relationships in training adaptation, suggesting the DRL system enhances outcomes primarily through superior timing and individualization rather than fundamentally novel training stimuli.

Training efficiency metrics are calculated as the ratio of performance improvement to training volume, demonstrating that athletes using the intelligent system achieve superior adaptation responses with reduced overall training volumes. The efficiency gains are particularly pronounced in endurance sports where the algorithm’s ability to optimize intensity distribution and recovery timing results in training efficiency improvements ranging from 1.38 to 1.42 compared to traditional periodization methods.

The injury prevention analysis demonstrates significant reductions in training-related injuries across all tested sports disciplines, with overall injury rate reductions of 43% compared to control groups using conventional training methods^55^. The intelligent system’s ability to monitor fatigue accumulation and adjust training loads proactively contributes to injury prevention through the identification of high-risk periods and automatic load adjustments. The fatigue index measurements, calculated as normalized physiological stress markers, consistently remain within optimal ranges (0.23–0.35) that minimize injury risk while maintaining sufficient training stimuli for continued adaptation.

Fatigue management effectiveness evaluation reveals enhanced recovery optimization and reduced cumulative fatigue through intelligent load distribution and recovery prescription^56^. The system’s real-time monitoring capabilities enable precise fatigue quantification and personalized recovery protocol implementation, resulting in average recovery time reductions of 23% compared to traditional methods. The fatigue management improvements are most pronounced in high-intensity sports where the algorithm’s ability to detect early fatigue indicators and adjust subsequent training loads prevents the accumulation of excessive physiological stress.

The psychological satisfaction assessment through standardized questionnaires demonstrates high athlete acceptance and perceived effectiveness of the intelligent training system across all tested sports^57^. Satisfaction scores ranging from 7.6 to 9.1 on a 10-point scale indicate strong athlete confidence in the system’s recommendations and perceived benefits in training quality and competitive preparation. The satisfaction analysis reveals that athletes particularly value the personalized approach and objective feedback provided by the intelligent system, contributing to improved training motivation and adherence rates.

The long-term effectiveness evaluation employed a multi-stage follow-up protocol to assess sustainability of system benefits over extended periods. The follow-up timeline consisted of three phases: short-term (0–16 weeks, primary intervention with weekly assessments), medium-term (4–6 months post-intervention with bi-weekly assessments), and long-term (6–24 months with monthly assessments). Assessment metrics included performance maintenance measured through standardized sport-specific tests repeated monthly and compared against baseline and post-intervention peak values. Adaptation sustainability was evaluated through performance trajectory slope analysis to identify continued improvement versus plateaus or decline. At 12 months, a 2-week system withdrawal test was conducted with a subset of athletes (*n* = 84, 28% of remaining sample) to assess performance dependency on continued algorithm use.

Statistical analysis utilized mixed-effects models for longitudinal data, controlling for individual athlete random effects and testing time×group interaction effects to determine effect persistence. Effect size decay rates were calculated to quantify diminishing returns over time. Of the original 338 participants, 284 (84%) completed the full 24-month follow-up, with attrition equally distributed across groups (AI: 82%, Control: 86%, χ²=0.89, *p* = 0.35). Dropout reasons included retirement (*n* = 21), team transfers (*n* = 18), and pregnancy/medical conditions (*n* = 15).

Results from the 24-month follow-up (*n* = 284) demonstrated that performance improvements relative to baseline were maintained at 78% of initial gains at 12 months (95% CI: 71–85%, *p* < 0.001 vs. control) and 62% at 24 months (95% CI: 54–70%, *p* = 0.003 vs. control). While absolute effect sizes declined over time, the AI group retained statistically significant advantages over controls throughout the follow-up period (12-month between-group difference: 9.6%, 95% CI: 7.2–12.0%; 24-month difference: 7.4%, 95% CI: 4.9–9.9%). Time×group interaction analysis revealed significant effects (F(23,6348) = 3.71, *p* < 0.001), indicating differential trajectories with AI group benefits gradually diminishing but remaining clinically meaningful.

The 2-week withdrawal test revealed an 8.2% (SD = 4.1%) performance decline when algorithmic guidance was removed, suggesting some degree of system dependency. However, 73% of athletes maintained performance above their original baseline despite withdrawal, indicating that acquired adaptations persisted partially independent of continued system use. Injury rates remained significantly lower in the AI group throughout follow-up (24-month cumulative incidence: AI 12.3% vs. Control 21.7%, HR = 0.61, 95% CI: 0.45–0.82, *p* < 0.001). Endurance sports demonstrated the strongest sustainability, with distance runners and swimmers maintaining 71% and 68% of initial gains at 24 months respectively, compared to 52–58% for technical and power sports. These findings support claims of long-term effectiveness while acknowledging gradual effect decay requiring periodic system updates and parameter recalibration to maintain optimal performance.

The comparative analysis between intelligent and traditional training methods reveals significant advantages in multiple performance dimensions, with effect sizes ranging from moderate (d = 0.6) to large (d = 1.2) across different evaluation metrics. The comprehensive statistical analysis confirms that the observed improvements are both statistically significant (*p* < 0.001) and practically meaningful for competitive athletics, representing performance gains that can influence competitive outcomes and long-term athletic development trajectories.

The economic efficiency assessment demonstrates that the performance improvements achieved through intelligent training load control provide substantial return on investment through reduced injury costs, improved competitive results, and enhanced training effectiveness. The cost-benefit analysis indicates that the system implementation costs are recovered within 8–12 months through performance gains and injury prevention benefits, making the technology economically viable for both individual athletes and sports organizations seeking competitive advantages through evidence-based training optimization.

### Limitations and potential confounding factors

While the results demonstrate strong associations between AI-assisted training and improved outcomes, several limitations and potential confounding factors must be acknowledged. First, the observed improvements cannot be unequivocally attributed to the DRL algorithm alone, as several confounders may have contributed to the results. The Hawthorne effect—wherein participants modify behavior due to awareness of being observed—may have increased training adherence in both groups, though potentially more so in the AI group given the novelty of the technology. Enhanced coach awareness resulting from structured system interaction may have independently improved load management practices beyond the algorithm’s direct contributions. Placebo effects, wherein athletes’ beliefs in technological superiority influence performance, cannot be ruled out in this unblinded design. Selection bias may also be present, as volunteers willing to participate in technology-based interventions may represent more motivated athlete populations.

Second, the cold-start problem presents a significant practical limitation, as new athletes require 2–4 weeks of data accumulation before the system achieves optimal performance. During this initial period, system recommendations may be less accurate, potentially compromising training effectiveness for athletes with limited time before competition. Third, data drift over extended periods (> 12 months) can degrade accuracy by 5–15% as physiological response patterns evolve and sensor calibration changes. This necessitates periodic model recalibration and retraining, adding to operational complexity. Fourth, the system’s reliance on high-end wearable sensors (total cost: $2,500-5,000 per athlete) severely limits accessibility in resource-constrained settings, potentially exacerbating performance gaps between well-funded and under-resourced programs.

Fifth, the 16-week intervention period, while representing a complete training macrocycle, may not capture long-term adaptation dynamics or identify delayed adverse effects. Although 12-month follow-up data suggest sustained benefits, validation beyond 24 months remains incomplete. Sixth, psychological impacts of automated decision-making on athlete autonomy and intrinsic motivation warrant consideration, as over-reliance on algorithmic recommendations may diminish self-awareness and decision-making capabilities. Finally, the single-blind design, while unavoidable given the intervention nature, introduces potential performance assessment bias despite blinded outcome evaluators.

Future research should employ more rigorous control designs including wait-list control groups, sham AI interventions (providing random rather than optimized recommendations), and fully blinded assessment protocols where feasible. Factorial designs could help disentangle algorithm effects from coach awareness and technology engagement effects. Additionally, qualitative research exploring athlete and coach experiences would provide valuable insights into the psychological and social dimensions of AI-assisted training that quantitative metrics alone cannot capture.

### System reliability and prospects for widespread application

The evaluation of system stability and reliability during long-term operational deployment demonstrates exceptional performance consistency across extended monitoring periods exceeding 24 months of continuous operation^58^. The reliability assessment employs comprehensive monitoring protocols that track system uptime, data processing accuracy, algorithm convergence stability, and user interaction responsiveness under varying operational conditions including peak usage periods, network connectivity fluctuations, and hardware resource constraints. The stability analysis reveals that the deep reinforcement learning system maintains consistent performance characteristics with minimal degradation over extended operational periods, demonstrating robust algorithmic foundations suitable for mission-critical athletic training applications.

**Table 9:** System reliability Assessment.

Metric category	Performance indicator	Measured value	Target threshold
System Uptime	Operational Availability	99.7%	> 99.5%
Algorithm Stability	Convergence Consistency	98.3%	> 95.0%
Processing Reliability	Data Accuracy Rate	99.1%	> 98.0%
Response Performance	Average Response Time	1.2 s	< 2.0 s
User Experience	Satisfaction Rating	8.4/10	> 8.0/10
Fault Recovery	Mean Time to Recovery	3.7 min	< 5.0 min
Data Integrity	Error Rate	0.08%	< 0.1%

As Table 9 shows, the system consistently exceeds established reliability thresholds across all critical performance indicators, demonstrating operational readiness for large-scale deployment in competitive sports environments.

The computational efficiency analysis reveals optimized resource utilization characteristics that enable scalable deployment across diverse hardware configurations and organizational scales^59^. Detailed computational performance metrics demonstrate that the full DRL system requires 1.2 ± 0.3ms per training prescription inference (measured across 10,000 prescription generations), substantially longer than linear regression baseline (0.1 ± 0.01ms) and rule-based systems (0.3 ± 0.05ms) but comparable to LSTM networks (0.8 ± 0.2ms), representing acceptable real-time performance for practical deployment where prescriptions are generated daily rather than requiring sub-millisecond latency. Initial model training required 18–24 h on NVIDIA RTX 3090 GPUs for sport-specific model convergence, considerably longer than LSTM baseline training (4–6 h) and linear regression (0.5–1 h), though this one-time training cost is amortized across subsequent months of operational use. The algorithm implementation demonstrates efficient memory management with peak memory usage of 2.5 ± 0.4GB during intensive processing periods, higher than LSTM (1.8 ± 0.3GB) and linear regression (0.3 ± 0.1GB) but within capabilities of standard server hardware. Model file sizes of 450 MB for the full DRL system exceed LSTM (180 MB) and linear regression (5 MB) baselines, impacting storage and deployment bandwidth but remaining manageable for cloud-based or local server implementations. CPU utilization maintains acceptable levels below 70% during real-time training load optimization tasks when processing prescriptions for up to 100 athletes simultaneously. The computational resource requirements scale linearly with the number of monitored athletes (R²=0.97 for memory usage vs. athlete count relationship), enabling cost-effective expansion from individual athlete monitoring to comprehensive team-based implementations without proportional increases in infrastructure complexity. Energy consumption during inference averaged 0.8 W per prescription generation, resulting in negligible operational electricity costs (<$50 annually for a 30-athlete team with daily prescriptions). These computational characteristics indicate that while the DRL system requires greater resources than simpler baselines, the requirements remain within practical bounds for organizations with standard IT infrastructure, and the performance benefits (12.3% vs. 5.4–8.7% for baselines) justify the computational overhead for competitive applications where marginal performance gains provide strategic advantages.

The resource consumption assessment indicates that the system operates effectively on standard computing hardware configurations, eliminating the need for specialized high-performance computing infrastructure^60^. Cloud-based deployment options provide additional scalability and cost optimization opportunities, particularly for organizations with variable athlete populations or seasonal training demands. The energy efficiency analysis demonstrates that the system’s power consumption remains minimal compared to traditional monitoring equipment, contributing to sustainable operational practices while maintaining superior analytical capabilities.

**Table 10:** Widespread application Cost-Benefit Analysis.

Application scenario	Implementation cost	Expected revenue	Technical difficulty	Personnel training	Maintenance cost	ROI period
Professional Teams	$150,000–200,000	$500,000–750,000	Medium	40 h	$30,000/year	8–12 months
Olympic Training Centers	$300,000–400,000	$800,000–1,200,000	High	80 h	$60,000/year	12–18 months
University Programs	$50,000–80,000	$150,000–300,000	Low-Medium	24 h	$15,000/year	6–10 months
Elite Individual Athletes	$25,000–40,000	$100,000–200,000	Low	16 h	$8,000/year	4–8 months
Youth Development	$30,000–50,000	$80,000–150,000	Low	20 h	$10,000/year	6–12 months

As Table 10 shows, the cost-benefit analysis demonstrates favorable return on investment across different competitive levels, with professional teams and Olympic training centers showing the highest absolute returns, while university programs and individual athletes benefit from lower implementation barriers and faster payback periods.

The prospects for widespread application across different competitive levels demonstrate significant potential for transformative impact on athletic training practices from grassroots to elite levels^61^. The implementation strategy analysis reveals that professional sports organizations represent the most immediate adoption targets due to their financial resources, technological infrastructure, and competitive pressures that drive innovation adoption. Olympic training centers and national sports institutes provide additional high-value deployment opportunities where the system’s performance optimization capabilities align with medal-winning objectives and long-term athlete development goals.

The scalability assessment for amateur and youth sports applications requires consideration of cost constraints, technical complexity, and organizational capacity limitations that may restrict full-feature implementation^62^. To empirically assess these implementation barriers, we conducted post-study surveys and interviews with coaches (*n* = 42), athletes (*n* = 338), and administrators (*n* = 15) across participating organizations using standardized questionnaires and semi-structured interviews. Regarding athlete autonomy and psychological impacts, athletes reported moderate reductions in perceived decision-making autonomy (mean reduction 2.3 points on 10-point scale, SD = 1.8), with 34% expressing concerns about over-reliance on algorithmic recommendations potentially diminishing their ability to interpret internal physiological signals independently. However, 78% simultaneously reported that algorithmic guidance enhanced their understanding of training principles and physiological responses through transparent explanations accompanying prescriptions. Technology acceptance was generally high (mean System Usability Scale score 76.2/100, indicating “good” usability), though 28% expressed concerns about data privacy, particularly regarding potential access by team management, sponsors, or competing organizations. These privacy concerns were more prevalent among professional athletes (42%) compared to collegiate athletes (18%), reflecting greater stakes in competitive advantage and commercial interests. Cost barriers emerged as the most significant impediment to wider adoption: 73% of amateur-level programs and 61% of youth development programs indicated that implementation costs ($50,000–200,000 for organizational deployment, $2,500-5,000 per athlete for sensors) were prohibitively expensive given typical budgets, compared to only 12% of professional organizations citing cost as a barrier. Administrators from lower-tier programs emphasized that sensor costs alone would consume 15–40% of annual equipment budgets, making adoption infeasible without external funding. Equity concerns were prominent, with 67% of respondents worried that technology-driven performance gaps would systematically advantage well-resourced programs, potentially violating competitive fairness principles, particularly problematic in youth sports where developmental opportunities should be equitable. Technical expertise requirements posed moderate barriers: 58% of organizations indicated they would require hiring data scientists or IT specialists (annual cost $60,000–100,000) to maintain systems, representing significant ongoing investment beyond initial deployment. Coach training time averaged 32 h (range 16–48 h) for achieving competency with system operation and interpretation, competing with existing professional development priorities. System dependency concerns were expressed by 45% of coaches who worried that algorithmic decision-making might deskill coaching expertise over time, particularly for less experienced coaches who might rely on recommendations rather than developing independent judgment. Despite these barriers, 82% of participants indicated willingness to continue using the system given access, and 71% believed benefits justified costs for competitive programs with adequate resources. Suggested solutions included developing tiered systems with reduced sensor requirements for budget-constrained contexts (estimated 70–80% effectiveness at 20% cost using smartphone-based monitoring), creating shared-resource models where multiple teams share sensor equipment and data infrastructure, establishing public funding programs or equipment subsidies for youth and amateur programs to promote equity, and developing open-source algorithm implementations to reduce licensing costs while maintaining core functionality.

Simplified system configurations designed for lower-tier applications can provide substantial training optimization benefits while maintaining affordable implementation costs and reduced technical complexity. The modular system architecture enables progressive capability expansion as organizations develop technical expertise and financial resources, creating sustainable adoption pathways across diverse competitive environments.

The implementation strategy recommendations emphasize phased deployment approaches that begin with proof-of-concept installations in high-visibility applications followed by systematic expansion based on demonstrated success and organizational learning. Training and support infrastructure development constitutes a critical component of successful widespread adoption, requiring comprehensive educational programs for coaches, sports scientists, and administrative personnel. The technology transfer strategy incorporates partnerships with sports equipment manufacturers, software developers, and educational institutions to facilitate knowledge dissemination and technical support across diverse implementation contexts.

The long-term sustainability of widespread adoption depends on continued algorithm development, hardware cost reductions, and integration with emerging sports technology ecosystems that enhance overall value propositions for end users. The system’s demonstrated reliability, proven performance benefits, and favorable cost-benefit characteristics establish strong foundations for transformative impact on competitive sports training practices across multiple organizational levels and competitive contexts.

### Ethical considerations and societal implications

The implementation of AI-driven training systems raises important ethical considerations regarding data privacy, access equity, and psychological well-being that warrant careful attention. Regarding data privacy, the continuous collection of physiological data creates significant privacy risks, as sensitive health information could be vulnerable to unauthorized access, misuse, or commercial exploitation. This study adhered to GDPR and HIPAA guidelines with all data encrypted during storage and transmission, accessible only to authorized research personnel. Athletes retained full data ownership rights and the ability to withdraw consent at any time. However, in commercial implementations, power imbalances between athletes and organizations may compromise true voluntariness of consent, particularly for athletes dependent on team selection or sponsorship. Robust data governance frameworks, including independent ethical oversight and transparent data usage policies, are essential to protect athlete rights.

Access equity presents another critical concern, as the substantial costs associated with high-end sensors ($2,500-5,000 per athlete) and computational infrastructure ($50,000–200,000 for organizational implementation) may exacerbate existing disparities between elite and amateur sports. This technology-driven performance gap could systematically advantage well-resourced programs while leaving under-resourced athletes further behind, potentially violating principles of fair competition. Development of low-cost sensor alternatives (<$500 per athlete) and open-source algorithm implementations could democratize access, though this requires sustained research investment and policy support. Sports governing bodies may need to establish regulations ensuring equitable access to performance-enhancing technologies.

Psychological impacts of automated decision-making also merit consideration. Over-reliance on algorithmic recommendations may diminish athlete self-awareness, intuition, and decision-making autonomy—capabilities that contribute to long-term development and psychological resilience. Coaches may experience deskilling if algorithms supplant rather than augment their expertise, potentially reducing coaching effectiveness in situations where technology is unavailable. The system should be positioned as a decision support tool that enhances rather than replaces human judgment, with explicit efforts to maintain athlete and coach agency. Education programs emphasizing critical evaluation of algorithmic recommendations and integration with experiential knowledge can help preserve human-centered decision-making.

Finally, the potential for algorithmic bias warrants attention. If training data predominantly represent certain demographic groups (e.g., male athletes, specific ethnic backgrounds), algorithms may perform suboptimally for underrepresented populations. This study’s participant demographics (72% male, predominantly Asian participants from one geographic region) limit generalizability. Future research should prioritize diverse training datasets and conduct fairness audits to identify and mitigate algorithmic bias. Transparent reporting of algorithm performance across demographic subgroups is essential for identifying disparities and ensuring equitable outcomes.

## Conclusion

This research presents an investigation into the application of deep reinforcement learning for personalized training load control in competitive sports, suggesting potential utility in adaptive athletic training optimization within well-resourced competitive environments. The core contribution of this study lies in the development of a deep reinforcement learning framework that integrates individual athlete physiological characteristics, real-time performance monitoring, and adaptive decision-making algorithms that shows associations with improved training load optimization compared to traditional methodologies^63^, though causal attribution is limited by study design constraints including single-blind methodology and potential confounding factors. The research establishes theoretical foundations through mathematical modeling of personalized training load relationships, fatigue-recovery dynamics, and multi-objective optimization frameworks that enable individual athlete adaptation while maintaining computational efficiency within well-resourced implementation contexts.

The technical contributions include the design of hybrid neural network architectures for processing heterogeneous physiological data streams, implementation of multi-agent collaborative optimization frameworks for team sport applications, and development of adaptive strategies for athlete-specific personalization. The practical application value is demonstrated through empirical validation across diverse competitive sports disciplines, revealing performance improvements averaging 12.3% (95% CI: 10.1–14.5%, *p* < 0.001) across tested applications, injury rate reductions of 43%, and training efficiency enhancements ranging from 1.15 to 1.42 times traditional methods. The system’s operational reliability characteristics, including 99.7% availability and sub-2-second response times, demonstrate feasibility for deployment in professional competitive environments with adequate technical infrastructure and financial resources. However, implementation costs ($50,000–200,000) and technical expertise requirements currently limit accessibility outside elite settings.

Despite these contributions, the research acknowledges several important limitations that constrain generalizability and practical implementation. First, the cold-start problem creates a 2–4 week initialization period during which new athletes must accumulate sufficient data before the system achieves optimal performance, potentially compromising training effectiveness for athletes with imminent competitions. Second, data drift over extended periods (> 12 months) can degrade model accuracy by 5–15% as physiological response patterns evolve and sensor calibration changes, necessitating periodic recalibration and retraining that add operational complexity. Third, long-term adaptation dynamics beyond 24 months remain inadequately validated, with potential risks of training stagnation or accumulated errors in chronic load management.

Fourth, the system’s dependence on high-end wearable sensors ($2,500-5,000 per athlete) severely limits accessibility in amateur, youth, and resource-constrained settings, potentially exacerbating performance inequalities between well-funded and under-resourced programs. Fifth, the single-blind study design (necessitated by intervention visibility) introduces potential assessment bias despite blinded outcome evaluators. Sixth, confounding factors including Hawthorne effects, enhanced coach awareness, and placebo effects cannot be fully disentangled from direct algorithm contributions, making definitive causal attribution challenging. Seventh, the participant sample (72% male, predominantly from one geographic region) limits generalizability to diverse athlete populations, raising concerns about potential algorithmic bias in underrepresented groups.

Eighth, psychological impacts of automated decision-making on athlete autonomy, intrinsic motivation, and long-term skill development warrant further investigation, as over-reliance on algorithmic recommendations may diminish self-awareness and independent decision-making capabilities. Ninth, algorithm effectiveness demonstrates substantial variation across sports disciplines (7.4–16.8% improvement range), suggesting that sport-specific architectures and reward functions may be necessary rather than a unified framework. Finally, the computational infrastructure requirements (specialized hardware, IT support, data management systems) demand technical expertise beyond typical coaching staff capabilities, creating implementation barriers even when financial resources are available.

Future research directions should focus on developing more robust transfer learning mechanisms that enable rapid adaptation to new sports disciplines and athlete populations with minimal historical data requirements^64^. We propose a phased development roadmap for transfer learning implementation: Phase 1 (0–6 months) involves developing cross-sport transfer learning frameworks using pre-training on datasets from 100 + athletes across multiple disciplines to establish generalizable feature representations. Phase 2 (6–12 months) focuses on testing transfer effectiveness in five new sports not included in original training, with target reduction of cold-start periods from 4 weeks to 1 week while maintaining > 80% of full-system performance. Phase 3 (12–18 months) aims to develop meta-learning algorithms enabling personalization within 24 h of deployment for new athletes. Preliminary transfer learning experiments conducted with 15 athletes across three new sports not included in the main study (rugby *n* = 5, volleyball *n* = 5, ice hockey *n* = 5) demonstrated that models pre-trained on related sports (rugby←soccer, volleyball←basketball, ice hockey←soccer) achieved 79.8% of full-system performance (mean improvement 9.8% vs. 12.3% for sport-specific training) within the first week of deployment compared to 4 weeks required when training from scratch. Performance improved to 88.2% of full-system effectiveness (10.9% improvement) by week 2 and reached 94.1% (11.6% improvement) by week 3 as athlete-specific data accumulated. Cold-start performance degradation was less severe for sports with similar physiological demands (rugby and soccer sharing high-intensity interval characteristics showed 83% first-week performance; volleyball and basketball sharing explosive power demands showed 81%; ice hockey showing more degradation at 75% reflecting greater biomechanical differences). Statistical analysis using paired t-tests showed significant improvement in cold-start performance with transfer learning compared to training from scratch (mean reduction in sub-optimal performance days: 21.4 days, 95% CI: 16.8–26.0, *p* < 0.001). These pilot results, while limited by small sample size and lack of long-term follow-up, support the feasibility of transfer learning approaches for accelerating deployment in new sports contexts, though larger-scale validation across more diverse sports and longer monitoring periods is required before operationalization. The success of transfer learning appears dependent on physiological and biomechanical similarity between source and target sports, suggesting that developing sport taxonomy frameworks and similarity metrics could optimize transfer learning strategies.

The integration of emerging technologies including miniaturized wearable sensors (<$500 per athlete), edge computing capabilities for real-time processing without cloud dependency, and conversational AI coaching interfaces presents opportunities for enhanced system accessibility and user experience. Partnerships with wearable device manufacturers to develop sports-specific sensor configurations could reduce costs while maintaining measurement quality. The development of federated learning approaches could enable collaborative algorithm improvement across multiple sports organizations while maintaining data privacy and competitive advantage considerations, allowing smaller organizations to benefit from aggregated knowledge without sharing sensitive individual athlete data.

Addressing sensor accessibility challenges represents a critical priority for democratizing AI-assisted training technology. Current system reliance on high-end wearables ($2,500-5,000 per athlete comprising Polar H10 heart rate monitors $90, Catapult Vector GPS units $2,500, Cosmed K5 metabolic analyzers $8,000, Optojump neuromuscular testing systems $15,000 institutional investment, and Delsys EMG systems $3,000) creates substantial barriers for amateur and youth sports contexts where such investments are prohibitive. Potential solutions to improve accessibility include: (1) developing smartphone-based monitoring protocols leveraging built-in accelerometers for activity tracking and camera-based heart rate detection through photoplethysmography, reducing per-athlete costs to approximately $0–100 (assuming athletes possess smartphones) while accepting 20–30% reduction in monitoring precision based on preliminary validation studies showing smartphone heart rate accuracy of *r* = 0.89 compared to chest strap gold standard *r* = 0.98; (2) establishing partnerships with consumer wearable manufacturers (Garmin, Fitbit, Apple, Polar) to create dedicated sports performance editions with enhanced sampling rates and data export capabilities at intermediate price points ($300–800), leveraging existing consumer device ecosystems and economies of scale; (3) exploring simplified sensor configurations monitoring only highest-impact variables (daily resting heart rate via smartphone or low-cost optical sensors $50–100, subjective fatigue ratings via mobile app questionnaires, training duration and GPS-derived intensity via smartphone tracking) with algorithm adaptations to maintain 70–80% of full-system performance based on reduced feature sets, demonstrating acceptable effectiveness in preliminary testing with 20 athletes over 8 weeks who achieved 8.8% performance improvement compared to 12.1% with comprehensive monitoring; (4) developing equipment sharing protocols for team-based implementations where 5–10 high-quality sensors rotate among 30–50 athletes during training sessions when continuous 24/7 monitoring is less critical than session-specific data, reducing per-athlete investment to $250–500 through shared infrastructure. Preliminary testing of the smartphone-based “lite” system version with 28 college athletes over 12 weeks achieved 72% of full-system performance benefits (8.9% improvement vs. 12.4% with comprehensive monitoring, *p* < 0.001 vs. control 1.2% improvement, effect size d = 0.87) at < 5% of implementation costs ($4,200 for 28-athlete cohort vs. $84,000 for full system), providing viable options for resource-constrained contexts willing to accept modest performance trade-offs for substantial cost savings. Cloud-based software-as-a-service business models with tiered pricing (basic tier $50/month per athlete providing smartphone integration, standard tier $150/month including wearable sensor rental, premium tier $300/month with comprehensive monitoring) could further improve accessibility by converting large upfront capital expenditures into manageable operational expenses while generating sustainable revenue for continued system development and support. Policy interventions may be necessary to prevent technology-driven widening of performance gaps: sports governing bodies could establish regulations ensuring equitable access to performance technologies through subsidized equipment programs for under-resourced organizations, equipment caps limiting sophisticated monitoring to preserve competitive balance, or open-source algorithm requirements ensuring core technology remains accessible despite proprietary sensor ecosystems. Public funding programs supporting technological adoption in youth development and amateur sports could promote equity while accelerating innovation adoption across broader participation bases. These multi-pronged approaches combining technological simplification, economic model innovation, and policy interventions represent essential strategies for extending DRL-based training optimization beyond elite contexts toward democratized access benefiting wider athlete populations.

The broader implications of this research extend beyond immediate training load optimization applications, establishing foundations for adaptive sports training systems that integrate performance analysis, injury prevention, and athlete development planning^65^. The demonstrated feasibility of deep reinforcement learning in sports training applications suggests potential for expansion into related domains including rehabilitation medicine, fitness optimization for general populations, and occupational performance enhancement in physically demanding professions, though each application would require domain-specific validation and adaptation.

The future trajectory of adaptive sports training technology will likely emphasize increased automation, enhanced personalization capabilities, and improved integration with existing sports science workflows. The continued advancement of artificial intelligence algorithms, combined with improvements in sensor technology and data processing capabilities, positions adaptive training load control systems as potentially valuable tools that may contribute to competitive sports preparation and athlete development practices. However, realizing this potential requires addressing current limitations including cold-start periods, data drift management, accessibility barriers, and ethical concerns regarding data privacy and equitable access. The established theoretical frameworks, validated implementation methodologies in controlled settings, and demonstrated performance benefits in well-resourced environments provide foundations for continued innovation, though widespread adoption will depend on successful cost reduction, simplification of technical requirements, and development of policies ensuring equitable access across diverse competitive contexts.

## Data Availability

The datasets generated and analyzed during the current study are not publicly available due to privacy and confidentiality agreements with participating athletes and sports organizations, and ethical considerations as detailed in the Ethics Statement. Anonymized datasets may be made available from the corresponding author on reasonable request and subject to appropriate ethical approval and data sharing agreements.

## References

[1] Bourdon, P. C. et al. Monitoring athlete training loads: consensus statement. *Int. J. Sports Physiol. Perform.* **12** (sup2), 161–S2 (2017). 28463642 10.1123/IJSPP.2017-0208

[2] Gabbett, T. J. The training-injury prevention paradox: should athletes be training smarter and harder? *Br. J. Sports Med.* **50** (5), 273–280 (2016). 26758673 10.1136/bjsports-2015-095788 PMC4789704

[3] Halson, S. L. Monitoring training load to understand fatigue in athletes. *Sports Med.* **44** (2), 139–147 (2014). 10.1007/s40279-014-0253-z PMC4213373 25200666

[4] Impellizzeri, F. M., Rampinini, E. & Marcora, S. M. Physiological assessment of aerobic training in soccer. *J. Sports Sci.* **23** (6), 583–592 (2005). 16195007 10.1080/02640410400021278

[5] Kiely, J. Periodization paradigms in the 21st century: evidence-led or tradition-driven? *Int. J. Sports Physiol. Perform.* **7** (3), 242–250 (2012). 22356774 10.1123/ijspp.7.3.242

[6] Meeusen, R. et al. *Prevention, diagnosis, and Treatment of the Overtraining Syndrome: Joint Consensus Statement of the European College of Sport Science and the American College of Sports Medicine* Vol. 45, 186–205 (Medicine & Science in Sports & Exercise, 2013). 1. 10.1249/MSS.0b013e318279a10a 23247672

[7] Mnih, V. et al. Human-level control through deep reinforcement learning. *Nature* **518** (7540), 529–533 (2015). 25719670 10.1038/nature14236

[8] Claudino, J. G. et al. Current approaches to the use of artificial intelligence for injury risk assessment and performance prediction in team sports: a systematic review. *Sports Medicine-Open*. **5** (1), 1–12 (2019). 31270636 10.1186/s40798-019-0202-3 PMC6609928

[9] Rein, R. & Memmert, D. Big data and tactical analysis in elite soccer: future challenges and opportunities for sports science. *SpringerPlus* **5** (1), 1–13 (2016). 27610328 10.1186/s40064-016-3108-2 PMC4996805

[10] Coutinho, D. et al. Typical weekly workload of under 15, under 17, and under 19 elite Portuguese football players. *J. Sports Sci.* **33** (12), 1229–1237 (2015). 25789549 10.1080/02640414.2015.1022575

[11] Akenhead, R. & Nassis, G. P. Training load and player monitoring in high-level football: current practice and perceptions. *Int. J. Sports Physiol. Perform.* **11** (5), 587–593 (2016). 26456711 10.1123/ijspp.2015-0331

[12] Bartlett, J. D., O’Connor, F., Pitchford, N., Torres-Ronda, L. & Robertson, S. J. Relationships between internal and external training load in team-sport athletes: evidence for an individualized approach. *Int. J. Sports Physiol. Perform.* **12** (2), 230–234 (2017). 27194668 10.1123/ijspp.2015-0791

[13] Sutton, R. S. & Barto, A. G. *Reinforcement Learning: an Introduction* (MIT Press, 2018).

[14] Arulkumaran, K., Deisenroth, M. P., Brundage, M. & Bharath, A. A. Deep reinforcement learning: A brief survey. *IEEE. Signal. Process. Mag.* **34** (6), 26–38 (2017).

[15] Puterman, M. L. *Markov Decision Processes: Discrete Stochastic Dynamic Programming* (Wiley, 2014).

[16] Saw, A. E., Main, L. C. & Gastin, P. B. Monitoring the athlete training response: subjective self-reported measures Trump commonly used objective measures: a systematic review. *Br. J. Sports Med.* **50** (5), 281–291 (2016). 26423706 10.1136/bjsports-2015-094758 PMC4789708

[17] Foster, C. et al. A new approach to monitoring exercise training. *J. Strength. Conditioning Res.* **15** (1), 109–115 (2001). 11708692

[18] McLaren, S. J., Coventry, E., Spears, I. R. & Weston, M. The relationships between internal and external measures of training load and intensity in team sports: a meta-analysis. *Sports Med.* **48** (3), 641–658 (2018). 29288436 10.1007/s40279-017-0830-z

[19] LeCun, Y., Bengio, Y. & Hinton, G. Deep learning. *Nature* **521** (7553), 436–444 (2015). 26017442 10.1038/nature14539

[20] Seshadri, D. R. et al. Wearable sensors for monitoring the internal and external workload of the athlete. *NPJ Digit. Med.* **2** (1), 1–18 (2019). 31372506 10.1038/s41746-019-0149-2 PMC6662809

[21] Buchheit, M. Monitoring training status with HR measures: do all roads lead to rome? *Front. Physiol.* **5**, 73 (2014). 24578692 10.3389/fphys.2014.00073 PMC3936188

[22] Banister, E. W., Calvert, T. W., Savage, M. V. & Bach, T. M. A systems model of training for athletic performance. *Australian J. Sports Med.* **7** (3), 57–61 (1975).

[23] Hellard, P. et al. Assessing the limitations of the banister model in monitoring training. *J. Sports Sci.* **24** (5), 509–520 (2006). 16608765 10.1080/02640410500244697 PMC1974899

[24] Busso, T. Variable dose-response relationship between exercise training and performance. *Med. Sci. Sports Exerc.* **35** (7), 1188–1195 (2003). 12840641 10.1249/01.MSS.0000074465.13621.37

[25] Skorski, S. & Abbiss, C. R. The manipulation of training load within the training process: a conceptual perspective. *Sports Med.* **47** (6), 1015–1024 (2017).

[26] Issurin, V. B. New horizons for the methodology and physiology of training periodization. *Sports Med.* **40** (3), 189–206 (2010). 20199119 10.2165/11319770-000000000-00000

[27] Van Hasselt, H., Guez, A. & Silver, D. Deep reinforcement learning with double q-learning. Proceedings of the AAAI Conference on Artificial Intelligence, 30(1). (2016).

[28] Tampuu, A. et al. Multiagent Cooperation and competition with deep reinforcement learning. *PLoS One* **12**(4), e0172395. (2017). 10.1371/journal.pone.0172395 PMC5381785 28380078

[29] Plews, D. J., Laursen, P. B., Stanley, J., Kilding, A. E. & Buchheit, M. Training adaptation and heart rate variability in elite endurance athletes: opening the door to effective monitoring. *Sports Med.* **43** (9), 773–781 (2013). 23852425 10.1007/s40279-013-0071-8

[30] Williams, R. J. Simple statistical gradient-following algorithms for connectionist reinforcement learning. *Mach. Learn.* **8** (3–4), 229–256 (1992).

[31] Bertsekas, D. P. *Reinforcement Learning and Optimal Control* (Athena Scientific, 2019).

[32] Goodfellow, I., Bengio, Y. & Courville, A. *Deep Learning* (MIT Press, 2016).

[33] Razavian, A. S., Azizpour, H., Sullivan, J. & Carlsson, S. CNN features off-the-shelf: an astounding baseline for recognition. Proceedings of the IEEE Conference on Computer Vision and Pattern Recognition Workshops, 806–813. (2014).

[34] Schaul, T., Quan, J., Antonoglou, I. & Silver, D. Prioritized experience replay. arXiv preprint arXiv:1511.05952. (2015).

[35] Lillicrap, T. P. et al. Continuous control with deep reinforcement learning. *ArXiv Preprint arXiv*: 1509.02971 (2015).

[36] Krizhevsky, A., Sutskever, I. & Hinton, G. E. Imagenet classification with deep convolutional neural networks. *Adv. Neural. Inf. Process. Syst.* **25**, 1097–1105 (2012).

[37] Bergstra, J. & Bengio, Y. Random search for hyper-parameter optimization. *J. Mach. Learn. Res.* **13** (2), 281–305 (2012).

[38] Kingma, D. P. & Ba, J. Adam: A method for stochastic optimization. arXiv preprint arXiv:1412.6980. (2014).

[39] Hartigan, J. A. & Wong, M. A. Algorithm AS 136: A k-means clustering algorithm. *J. Roy. Stat. Soc.: Ser. C (Appl. Stat.)*. **28** (1), 100–108 (1979).

[40] Finn, C., Abbeel, P. & Levine, S. Model-agnostic meta-learning for fast adaptation of deep networks. International Conference on Machine Learning, 1126–1135. (2017).

[41] Bergmeir, C. & Benítez, J. M. On the use of cross-validation for time series predictor evaluation. *Inf. Sci.* **191**, 192–213 (2012).

[42] Thorpe, R. T. et al. Monitoring fatigue during the in-season competitive phase in elite soccer players. *Int. J. Sports Physiol. Perform.* **10** (8), 958–964 (2015). 25710257 10.1123/ijspp.2015-0004

[43] Hopkins, W. G., Marshall, S. W., Batterham, A. M. & Hanin, J. Progressive statistics for studies in sports medicine and exercise science. *Med. Sci. Sports Exerc.* **41** (1), 3–13 (2009). 19092709 10.1249/MSS.0b013e31818cb278

[44] Laursen, P. B. & Jenkins, D. G. The scientific basis for high-intensity interval training. *Sports Med.* **32** (1), 53–73 (2002). 11772161 10.2165/00007256-200232010-00003

[45] Seiler, S. What is best practice for training intensity and duration distribution in endurance athletes? *Int. J. Sports Physiol. Perform.* **5** (3), 276–291 (2010). 20861519 10.1123/ijspp.5.3.276

[46] Spencer, M., Bishop, D., Dawson, B. & Goodman, C. Physiological and metabolic responses of repeated-sprint activities. *Sports Med.* **35** (12), 1025–1044 (2005). 16336007 10.2165/00007256-200535120-00003

[47] Soligard, T. et al. How much is too much? (Part 1) international olympic committee consensus statement on load in sport and risk of injury. *Br. J. Sports Med.* **50** (17), 1030–1041 (2016). 27535989 10.1136/bjsports-2016-096581

[48] Gabbett, T. J. et al. The athlete monitoring cycle: a practical guide to interpreting and applying training monitoring data. *Br. J. Sports Med.* **51** (20), 1451–1452 (2017). 28646100 10.1136/bjsports-2016-097298

[49] Schulz, K. F., Altman, D. G. & Moher, D. CONSORT 2010 statement: updated guidelines for reporting parallel group randomised trials. *J. Clin. Epidemiol.* **63** (8), 834–840 (2010). 20346629 10.1016/j.jclinepi.2010.02.005

[50] Cohen, J. *Statistical Power Analysis for the Behavioral Sciences* (Lawrence Erlbaum Associates, 1988).

[51] Buchheit, M. & Laursen, P. B. High-intensity interval training, solutions to the programming puzzle. *Sports Med.* **43** (5), 313–338 (2013). 23539308 10.1007/s40279-013-0029-x

[52] Twist, C. & Highton, J. Monitoring fatigue and recovery in rugby league players. *Int. J. Sports Physiol. Perform.* **8** (5), 467–474 (2013). 23319463 10.1123/ijspp.8.5.467

[53] Kellmann, M. et al. Recovery and performance in sport: consensus statement. *Int. J. Sports Physiol. Perform.* **13** (2), 240–245 (2018). 29345524 10.1123/ijspp.2017-0759

[54] Coggan, A. R. & Allen, H. *Training and Racing with a Power Meter* (Velo, 2010).

[55] Windt, J. & Gabbett, T. J. How do training and competition workloads relate to injury? The workload—injury aetiology model. *Br. J. Sports Med.* **51** (5), 428–435 (2017). 27418321 10.1136/bjsports-2016-096040

[56] Nédélec, M. et al. Recovery in soccer. *Sports Med.* **42** (12), 997–1015 (2012). 23046224 10.2165/11635270-000000000-00000

[57] Davis, F. D. Perceived usefulness, perceived ease of use, and user acceptance of information technology. *MIS Q.* **13** (3), 319–340 (1989).

[58] Avizienis, A., Laprie, J. C., Randell, B. & Landwehr, C. Basic concepts and taxonomy of dependable and secure computing. *IEEE Trans. Dependable Secur. Comput.* **1** (1), 11–33 (2004).

[59] Hennessy, J. L. & Patterson, D. A. *Computer Architecture: a Quantitative Approach* (Morgan Kaufmann, 2019).

[60] Koomey, J., Berard, S., Sanchez, M. & Wong, H. Implications of historical trends in the electrical efficiency of computing. *IEEE Ann. Hist. Comput.* **33** (3), 46–54 (2011).

[61] Rogers, E. M. *Diffusion of Innovations* (Free, 2003).

[62] Venkatesh, V., Morris, M. G., Davis, G. B. & Davis, F. D. User acceptance of information technology: toward a unified view. *MIS Q.* **27** (3), 425–478 (2003).

[63] Silver, D. et al. Mastering the game of go with deep neural networks and tree search. *Nature* **529** (7587), 484–489 (2016). 26819042 10.1038/nature16961

[64] Pan, S. J. & Yang, Q. A survey on transfer learning. *IEEE Trans. Knowl. Data Eng.* **22** (10), 1345–1359 (2009).

[65] Russell, S. & Norvig, P. *Artificial Intelligence: a Modern Approach* (Pearson, 2020).

[66] Rossi, A. et al. Effective injury forecasting in soccer with GPS training data and machine learning. *PLoS ONE* **13**(7), e0201264 (2018). 10.1371/journal.pone.0201264 PMC6059460 30044858

[67] Teixeira, J. E. et al. Monitoring accumulated training and match load in football: A systematic review. *Int. J. Environ. Res. Public Health*. **18** (8), 3906 (2021). 33917802 10.3390/ijerph18083906 PMC8068156

[68] Jauhiainen, S. et al. New machine learning approach for detection of injury risk factors in young team sport athletes. *Int. J. Sports Med.* **42** (2), 175–182 (2021). 32920800 10.1055/a-1231-5304

[69] Sarlis, V., Papageorgiou, G. & Tjortjis, C. Sports analytics for evaluating injury impact on NBA performance. *Information* **16** (8), 699. 10.3390/info16080699 (2025).

[70] Iatropoulos, D., Sarlis, V. & Tjortjis, C. A Data Mining Approach to Identify NBA Player Quarter-by-Quarter Performance Patterns. *Big Data Cogn. Comput.*, **9**(4), 74. 10.3390/bdcc9040074 (2025).

[71] Sarlis, V., Gerakas, D. & Tjortjis, C. A Data Science and Sports Analytics Approach to Decode Clutch Dynamics in the Last Minutes of NBA Games. *Mach. Learn. Knowl. Extr.*, **6**(3), 2074–2095. 10.3390/make6030102 (2024).

[72] Gilgen-Ammann, R., Schweizer, T. & Wyss, T. RR interval signal quality of a heart rate monitor and an ECG Holter at rest and during exercise. *Eur. J. Appl. Physiol.* **119** (7), 1525–1532 (2019). 31004219 10.1007/s00421-019-04142-5

[73] Tanner, R. K., Fuller, K. L. & Ross, M. L. Evaluation of three portable blood lactate analysers: lactate Pro, lactate scout and lactate plus. *Eur. J. Appl. Physiol.* **109** (3), 551–559 (2010). 20145946 10.1007/s00421-010-1379-9

[74] Perez, M. et al. Validation of the cosmed K4b2 portable metabolic system. *Int. J. Sports Med.* **23** (3), 192–197 (2019).

[75] Glatthorn, J. F. et al. Validity and reliability of Optojump photoelectric cells for estimating vertical jump height. *J. Strength. Conditioning Res.* **25** (2), 556–560 (2011). 10.1519/JSC.0b013e3181ccb18d 20647944

[76] Hermens, H. J., Freriks, B., Disselhorst-Klug, C. & Rau, G. Development of recommendations for SEMG sensors and sensor placement procedures. *J. Electromyogr. Kinesiol.* **10** (5), 361–374 (2000). 11018445 10.1016/s1050-6411(00)00027-4

[77] Buysse, D. J., Reynolds, C. F., Monk, T. H., Berman, S. R. & Kupfer, D. J. The Pittsburgh sleep quality index: a new instrument for psychiatric practice and research. *Psychiatry Res.* **28** (2), 193–213 (1989). 2748771 10.1016/0165-1781(89)90047-4

[78] Scott, M. T., Scott, T. J. & Kelly, V. G. The validity and reliability of global positioning systems in team sport: A brief review. *J. Strength. Conditioning Res.* **30** (5), 1470–1490 (2016). 10.1519/JSC.0000000000001221 26439776

[79] Bijur, P. E., Silver, W. & Gallagher, E. J. Reliability of the visual analog scale for measurement of acute pain. *Acad. Emerg. Med.* **8** (12), 1153–1157 (2001). 11733293 10.1111/j.1553-2712.2001.tb01132.x

[80] Pearson, T. A. et al. Markers of inflammation and cardiovascular disease: application to clinical and public health practice. *Circulation* **107** (3), 499–511 (2003). 12551878 10.1161/01.cir.0000052939.59093.45

[81] Fuller, C. W. et al. Consensus statement on injury definitions and data collection procedures in studies of football (soccer) injuries. *Br. J. Sports Med.* **40** (3), 193–201 (2006). 16505073 10.1136/bjsm.2005.025270 PMC2491990

